# Predicting Daily Cardiovascular Emergencies Using Weather and Air Quality Data: A 23‐Year Machine‐Learning Analysis in Taiwan

**DOI:** 10.1029/2025GH001769

**Published:** 2026-06-12

**Authors:** Hsiang‐Han Chen, Pei‐Shan Tsai, Yu‐Chia Chen, Cheng‐Yu Li, Yu‐Kai Lin, Wan‐Ru Huang, Kate Huihsuan Chen

**Affiliations:** ^1^ Department of Computer Science and Information Engineering National Taiwan Normal University Taipei Taiwan; ^2^ Department of Earth Sciences National Taiwan Normal University Taipei Taiwan; ^3^ The Department of Health and Welfare University of Taipei Taipei Taiwan

**Keywords:** cardiovascular disease, meteorological variable, air quality variable, machine learning, CVD prediction, environmental health

## Abstract

Short‐term variability in weather and air quality is known to influence cardiovascular emergencies, yet its day‐to‐day predictive value at the population level remains insufficiently understood. Using 23 years of nationwide data from Taiwan (2000–2022), we evaluated how weather and air quality conditions shape daily cardiovascular disease (CVD) emergency visits across geographic regions. We first applied unsupervised learning methods, including UMAP and K‐means clustering, to 184 environmental features to identify data‐driven environmental regimes and examine the distribution of high‐risk CVD days. We then trained eight supervised learning models to predict daily CVD emergency visits and used SHAP values to interpret key predictors. Unsupervised analyses revealed consistent seasonal and pollution‐related patterns. High‐risk days tended to cluster during cool conditions accompanied by elevated air pollution, with temperatures higher than in winter but substantially greater pollution levels. This pattern was particularly evident in northern Taiwan and among populations aged 65 years and older. Air‐pollution variables produced more clearly defined high‐risk clusters than meteorological variables alone, indicating a stronger pollution‐related contribution to acute CVD risk. In the supervised framework, tree‐based ensemble models (Random Forest, LightGBM, XGBoost) achieved the best performance, with *R* ^2^ values up to 0.67 and mean absolute percentage errors of 7%–8%. Predictability was highest for elderly populations and northern Taiwan. SHAP analyses identified NO_x_‐related metrics as dominant predictors of CVD emergency visits. These results demonstrate that high‐resolution environmental data, combined with machine‐learning methods, can effectively predict CVD emergencies, delineate high‐risk environmental regimes, and support region‐specific early‐warning systems centered on air‐pollution monitoring, particularly NO_x_.

## Plain Language Summary

Weather changes and air pollution can affect heart health, but it remains difficult to predict which specific combinations of conditions lead to more people needing emergency care. In this study, we analyzed 23 years of nationwide data from Taiwan to understand how daily weather and air quality conditions relate to cardiovascular disease (CVD) emergency visits. Using machine‐learning methods, we identified a distinct environmental pattern: days that were relatively cool but had high levels of air pollution, especially nitrogen oxides (NO_x_) that were linked to notably higher CVD emergency visits, particularly among older adults. We also developed prediction models that can estimate daily CVD emergency visits with good accuracy. Overall, our results show that air pollution, more than temperature alone, plays a major role in triggering cardiovascular emergencies. Monitoring air pollution, especially NO_x_, could help health agencies and communities prepare earlier and better protect vulnerable populations.

## Key Points

- High‐risk cardiovascular disease (CVD) days occur during cool but highly polluted environmental conditions
- Air‐pollution variables, especially NO_x_, define high‐risk clusters better than other weather factors
- Machine‐learning models accurately predict daily CVD emergency visits nationwide

## 1 Introduction

Cardiovascular diseases (CVDs) are among the leading causes of morbidity and mortality worldwide, responsible for nearly 20 million deaths every year (Cesare et al., 2024; Wang et al., 2024). Rapid urbanization, population aging, and environmental stress have intensified the burden of cardiovascular emergencies across East Asia (Goh et al., 2024). The onset and progression of CVDs result from the interaction of intrinsic and extrinsic determinants. Intrinsic factors include genetic predisposition, hypertension, diabetes, obesity, hyperlipidemia, and behavioral risks such as smoking, drinking, and physical inactivity (Banerjee & Asrress, 2018; van Bussel et al., 2020). Age and gender remain dominant demographic predictors, as older adults and males consistently exhibit higher hospitalization and mortality rates (Merz & Cheng, 2016; Mikkola et al., 2013; Rodgers et al., 2019). Pre‐existing diabetes mellitus, whether type 1 or type 2, further amplifies cardiovascular vulnerability, leading to increased event frequency and mortality across diverse populations (Dal Canto et al., 2019; Rawshani et al., 2019; Strain & Paldánius, 2018).

Beyond intrinsic personal health characteristics, environmental exposures can acutely precipitate cardiovascular events. Particulate matter (PM_2.5_ and PM_10_), nitrogen dioxide (NO_2_), carbon monoxide (CO), ozone (O_3_), and meteorological extremes are consistently linked to elevated CVD risk (Al‐Kindi et al., 2020; Moghadamnia et al., 2017; O’Toole et al., 2008). These effects are mediated by oxidative stress, systemic inflammation, and endothelial dysfunction and are often amplified in dense urban or socially vulnerable communities (Cosselman et al., 2015; Malambo et al., 2016; Münzel et al., 2017; Shrivastav et al., 2024). Understanding the contribution of environmental triggers is therefore essential for comprehensive CVD prevention and public‐health planning.

Taiwan provides an informative natural laboratory for examining the environmental predictability of CVD outcomes. This island's steep climatic and geographic gradients, from humid subtropical basins in the north to tropical plains in the south and high mountain regions inland (Huang et al., 2018), produce diverse combinations of temperature, humidity, and pollution regimes within a compact land area (Fang et al., 2025; C.‐Y. Lin et al., 2021). This heterogeneity offers a unique opportunity to evaluate how short‐term environmental variability influences acute cardiovascular events across distinct demographic and regional contexts.

CVDs consistently rank among the top five causes of death in Taiwan, and hospital admissions for acute cardiac events have increased steadily over the past two decades (Chang et al., 2005; Liang et al., 2009; D.‐H. Tsai et al., 2010; Yen et al., 2024). Previous studies have demonstrated short‐term increases in CVD admissions associated with elevated PM_2.5_, PM_10_, NO_2_, and CO concentrations (Chang et al., 2005; Liang et al., 2009; D.‐H. Tsai et al., 2010; Yen et al., 2024). Early work in Taipei showed that daily CVD admissions correlated with PM_10_, NO_2_, and CO concentrations, with clear temperature dependencies: particulate matter dominated under cooler conditions, whereas gaseous pollutants were more influential during warmer periods (Chang et al., 2005). Y.‐K. Lin et al. (2020) further demonstrated that disease‐risk patterns differ geographically: cooler temperatures in northern Taiwan elevate ischemic heart‐disease risk, whereas warmer conditions exert comparable influence in the south. More recent analyses demonstrated that reductions in traffic and industrial activity during the COVID‐19 pandemic coincided with lower PM levels and a measurable decline in acute myocardial infarction and heart‐failure hospitalizations (Yen et al., 2024). Yet, most investigations remain city‐specific or limited to short temporal windows. Important questions remain regarding regional variability, demographic heterogeneity, and predictability across Taiwan.

To address this gap, the present study integrates 23 years (2000–2022) of nationwide data to quantify and predict daily cardiovascular emergency visits in relation to environmental variability. We employ a suite of machine‐learning methods to identify the most influential predictors and to assess spatial and demographic differences in environmental sensitivity. Building on advances in environmental data integration and prediction modeling, we ask three key questions: (1) To what extent can daily CVD emergency visits be predicted from environmental factors? (2) Which demographic groups (by age, gender, or region) show the highest environmental predictability? (3) Which environmental variables most strongly influence the short‐term occurrence of acute cardiovascular events? To address these questions, we combine the CVD emergency visit records from Taiwan's National Health Insurance Research Database (NHIRD) with continuously daily meteorological and air quality measurements from Taiwan's monitoring networks. This integrated analysis aims to advance data‐driven forecasting of CVD emergencies and to inform region‐specific early‐warning system under evolving weather and air quality conditions.

## 2 Materials and Methods

### 2.1 Study Area

**Figure 1:**
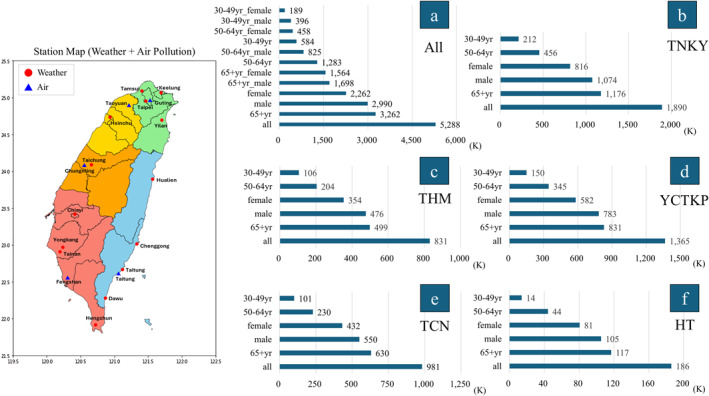
Study regions, monitoring stations, and cardiovascular emergency‐visit counts in Taiwan. Left panel: Geographic division of Taiwan into five regions—Northern (TNKY; green), Northwestern (THM; yellow), Central (TCN; orange), Southern (YCTKP; red), and Eastern (HT; blue). Red circles indicate representative weather stations from Taiwan's Central Weather Administration, and blue triangles denote air‐quality monitoring stations from the Environmental Protection Administration (now Ministry of Environment). Right panel (a–f): Total CVD–related emergency visits from 2000 to 2022, stratified by sex (male, female) and age group (30–49, 50–64, ≥65 years). Panel (a) shows nationwide totals (all regions combined), and (b–f) show regional subsets for northern (TNKY), northwestern (THM), southern (YCTKP), central (TCN), and eastern (HT) Taiwan, respectively.

Taiwan was divided into five administrative–climatological regions to capture spatial heterogeneity in environmental conditions and population structure (Figure 1, left panel): northern Taiwan (TNKY: Taipei, New Taipei, Keelung, Yilan), northwestern Taiwan (THM: Taoyuan, Hsinchu, Miaoli), central Taiwan (TCN: Taichung, Changhua, Nantou), southern Taiwan (YCTKP: Yunlin, Chiayi, Tainan, Kaohsiung, Pingtung), and eastern Taiwan (HT: Hualien, Taitung). These regions reflect major climatological zones and demographic contrasts. Representative meteorological and air‐quality monitoring stations were selected near urban centers with relatively high population density so that environmental variables closely correspond to the spatial distribution of health data.

### 2.2 Environmental Data—Meteorological and Air Quality Variable

Meteorological and air quality data were obtained from nationwide monitoring networks for the period 2000–2022 and harmonized with daily CVD data for each of the five regions (TNKY, THM, TCN, YCTKP, HT, see monitoring station locations in Figure 1, left panel). Meteorological variables from the Central Weather Administration (CWA) of Taiwan included surface pressure (PS), wind speed (WS), precipitation (PP), relative humidity (RH), feel‐like temperature (FLT), and temperature (Temp). Air quality variables from the Environmental Protection Administration (EPA) monitoring networks included PM_2.5_, PM_10_, O_3_, SO_2_, CO, NO, NO_2_, and NO_x_. All variables were originally recorded at hourly resolution. After quality control and station‐level averaging, data were aggregated into daily regional values.

To derive features that capture daily fluctuations in environmental conditions, we constructed a family of descriptors for each meteorological and air‐pollution variable, representing absolute levels, short‐term variability, and diurnal structure. These descriptors included the daily mean (e.g., ps_avg for daily mean pressure), daily maximum and minimum values, and the daily range (max–min). To represent day‐to‐day changes, we calculated differences in the daily mean, maximum, minimum, and range relative to the previous day. Diurnal asymmetry was quantified using daytime (06:00–18:00) versus nighttime (18:00–06:00) contrasts in mean, maximum, minimum, and range, along with their day‐to‐day changes. For precipitation, only the daily total rainfall (PP_sum) was used. For relative humidity, we additionally computed DeHumidify_Load, defined as the daily average deviation above a comfort threshold of 70% relative humidity, representing the mean excess humidity during hours when RH exceeded 70%.

In total, 184 environmental features were generated for each daily sample in the 23‐year data set. These features characterize both broad climatic conditions and short‐term pollution‐related perturbations, enabling the machine‐learning models to account for both nonlinear and transient environmental influences on cardiovascular risk.

### 2.3 Cardiovascular Emergency‐Visit Data

Daily emergency‐visit records were obtained from NHIRD for the 23‐year period from 2000 to 2022. CVD‐related visits were identified using ICD‐9 codes 410–414, 430–438, 401–405, 428, and 440–448 and aggregated by region, sex, and age group. This large‐scale data set provides comprehensive national coverage for examining the relationship between daily CVD incidence and environmental variability.

During the 23‐year period, the total number of emergency visits were recorded 4.038 million per year in average across all diseases. CVD‐related visits accounted for 5.7% of total emergency visits, corresponding to an annual average of approximately 230,000 CVD cases. The health data set was stratified into two sex categories (male, female), three age groups (30–49 years, 50–64 years, and ≥65 years), and the five geographic regions defined in Section 2.1.

Figure 1 (right panel) summarizes the total number of CVD‐related emergency visits (5.28 million) across Taiwan between 2000 and 2022, classified by geographic region, age group, and sex. Older adults (≥65 years) represented the largest proportion of cases (about 62%), followed by adults aged 50–64 years (24%) and those aged 30–49 years (14%). This age distribution was consistent across regions. Gender differences were also pronounced. Male accounted for 57% of all CVD emergency visits nationwide, with the greatest difference in the 50–64‐year and ≥65‐year groups, suggesting a higher CVD vulnerability or care‐seeking frequency among men. Regionally, the northern region (TNKY) had the highest visit counts (about 1.89 million), followed by southern Taiwan (YCTKP; about 1.36 million) and central Taiwan (TCN; ≈0.98 million). The northwestern (THM) and eastern (HT) regions contributed smaller totals, consistent with their population sizes. After normalizing by population, eastern Taiwan exhibited the highest per‐capita CVD emergency rates, due to demographic aging and limited medical accessibility. Despite regional contrasts, the consistent demographic pattern appears across Taiwan: older men accounted for the largest proportion of cardiovascular emergency visits, whereas younger women had the fewest.

To preliminarily assess environmental influence, we first compared the seasonal cycles of each meteorological and air quality variable with seasonal patterns in CVD emergency visits (see Figure S1 in Supporting Information S1 for meteorological variables and Figure S2 in Supporting Information S1 for air quality variables). These analyses showed that cold, dry, and low‐precipitation winter conditions are associated with the highest CVD emergency incidence, whereas warm, humid, and rainy summer conditions coincide with substantially lower CVD occurrences, consistent with seasonal CVD risk patterns observed in temperate and subtropical climates (Stewart et al., 2017). Air pollution showed a similar seasonal pattern, with concentrations and higher CVD incidence in winter. However, these relationships involve multiple interacting variables that covary nonlinearly. To quantify the individual and combined effects, we subsequently applied machine learning models that incorporate the full set of meteorological and air‐pollution predictors.

### 2.4 CVD Emergency‐Visit Prediction and Environmental Factor Analysis Using Machine Learning Methods

Machine learning methods were used to model the complex, nonlinear relationships between high‐dimensional environmental conditions and cardiovascular risk. By jointly considering multiple interacting meteorological and air‐pollution variables, these approaches can capture patterns that are difficult to detect with traditional linear or pairwise analyses and directly associate them with daily CVD emergency‐visit rates. Specifically, we combined unsupervised learning to explore data structure and risk‐associated patterns with supervised regression models to quantify predictive performance and identify influential environmental drivers across regions and demographic groups. For all analyses, the target variable was the daily CVD emergency‐visit rate, calculated as the number of CVD emergency visits divided by the total number of emergency visits for all diseases on the same day for each region, sex, and age group. This normalization accounts for day‐to‐day fluctuations in overall emergency department utilization, allowing for comparison of the relative burden of CVD visits across days rather than relying on raw counts or population‐based rates.

#### 2.4.1 Data Analysis and Visualization Using Unsupervised Learning Methods

First, unsupervised learning methods were employed to elucidate the relationships between environmental factors and the CVD emergency‐visit rate across different regions and demographic groups. To visualize the high‐dimensional environmental data, we applied the Uniform Manifold Approximation and Projection (UMAP) algorithm (McInnes et al., 2020) to project environmental features into a two‐dimensional space while preserving the intrinsic structure of the original data, particularly the local relationships among samples. The resulting UMAP embeddings were then used to visually examine associations between environmental factors and CVD emergency‐visit rates and to explore potential patterns across population subgroups.

To quantitatively assess the relationship between environmental features and CVD emergency‐visit rates across different groups, we implemented an unsupervised clustering procedure. Specifically, the K‐means algorithm was applied to partition samples into two clusters based solely on environmental features. The number of clusters was fixed at two to distinguish between putative high‐ and low‐risk groups. Although the number of clusters in K‐means can be determined using data‐driven criteria (e.g., the elbow method), the choice of *k* = 2 was specified a priori to directly test whether environmental conditions could separate days into relatively high‐ and low‐risk groups. We then evaluated the proportion of high‐risk samples, defined as days with a CVD emergency‐visit rate exceeding the mean rate within each group, in each cluster. A significantly higher proportion of high‐risk samples in one cluster compared with the other would suggest that the clustering pattern is associated with CVD risk. Conversely, if high‐risk samples are randomly distributed between clusters, this would indicate a lack of association between environmental factors and CVD risk. The statistical significance of the proportional difference between clusters was evaluated using the chi‐square test.

#### 2.4.2 CVD Emergency‐Visit Rate Prediction Using Supervised Learning Models

To assess the predictability of daily CVD emergency visits and to identify the most influential environmental drivers, we comprehensively implemented eight supervised machine‐learning algorithms that are widely applied across various research domains. These include Ridge Regression (Ridge) (Hoerl & Kennard, 1970), Least Absolute Shrinkage and Selection Operator (LASSO) (Tibshirani, 1996), Support Vector Regression (SVR) (Vapnik, 1997), k‐Nearest Neighbors (KNN) (Cover & Hart, 1967), Multilayer Perceptron (MLP) (Rumelhart et al., 1986), Random Forest (RF) (Breiman, 2001), Light Gradient Boosting Machine (LightGBM) (Ke et al., 2017), and eXtreme Gradient Boosting (XGBoost) (Chen & Guestrin, 2016). These models collectively represent a broad spectrum of learning paradigms.

Ridge and LASSO are linear models with L2 and L1 regularization, respectively, which constrain model coefficients to reduce overfitting and enhance generalization. SVR is a kernel‐based regression method that employs nonlinear kernel functions to capture complex feature relationships; in this study, the radial basis function kernel was adopted. KNN is a nonparametric algorithm that predicts outcomes based on the mean response of the *k* nearest samples in the feature space, making it simple yet effective for local pattern recognition. MLP is a feedforward neural network that learns hierarchical nonlinear mappings through multiple hidden layers, enabling it to model intricate interactions among environmental features. RF is an ensemble learning method that constructs multiple decision trees using bootstrap samples and random feature subsets, and aggregates their predictions to reduce variance and improve robustness. LightGBM and XGBoost are gradient boosting frameworks that sequentially build ensembles of decision trees by minimizing residual errors in previous iterations; both methods are efficient, capable of handling large‐scale and high‐dimensional data. All models were implemented in Python using the scikit‐learn 1.7.2 library, with default parameter settings applied to provide a general assessment of model performance.

Collectively, these algorithms encompass distinct modeling philosophies including linear regularization, kernel‐based regression, neural representation learning, and ensemble decision trees. This diversity allows a comprehensive evaluation of CVD emergency rate prediction across different regression technologies and high‐dimensional environmental data sets.

#### 2.4.3 Evaluation and Interpretation of Supervised Learning Models

All supervised learning models were trained using 80% of the data set and independently tested on the remaining 20% to evaluate predictive generalization. A random split was adopted because the objective of the supervised analysis was to assess the overall association between environmental features and CVD emergency‐visit rates rather than to develop a temporal forecasting model. Performance was assessed using two standard quantitative metrics (a) Coefficient of Determination (*R* ^2^): measures the proportion of variance in observed CVD emergency visits explained by the model, providing an indicator of predictive strength. (b) Mean Absolute Percentage Error (MAPE): quantifies the average absolute difference between predicted and observed values relative to the observed values, reflecting overall prediction accuracy in percentage terms. These complementary metrics allow comparison across models of differing complexity. A higher *R* ^2^ and lower MAPE indicate stronger and more reliable predictive capability.

To further interpret the influence of individual predictors, we applied Shapley Additive Explanations (SHAP) (Lundberg & Lee, 2017) to the supervised learning models. SHAP decomposes each model prediction into additive feature contributions, allowing transparent interpretation of how meteorological and air‐pollution variables affect the predicted outcomes. This method identifies not only the most influential features but also the direction and magnitude of their effects, thereby bridging the gap between black‐box predictions and physically interpretable environmental insights.

## 3 Results

### 3.1 Environmental Factor Analysis Across Taiwan Using Unsupervised Learning Methods

**Figure 2:**
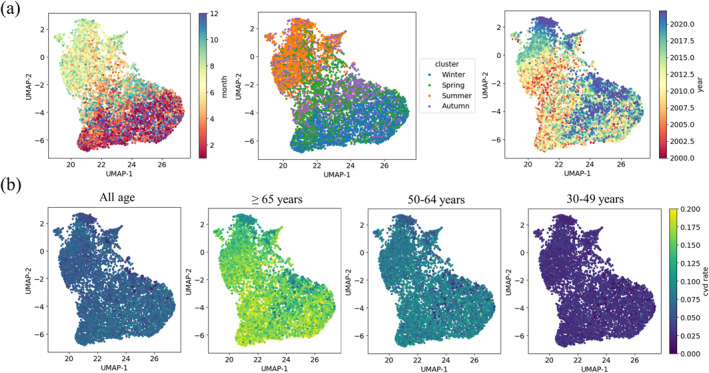
UMAP visualization of environmental features and CVD emergency‐visit rates across Taiwan. (a) UMAP projections of averaged environmental features across Taiwan, colored by month, season, and year (from left to right). (b) UMAPs colored by CVD emergency‐visit rates for all individuals, ≥65 years, 50–64 years, and 30–49 years (from left to right).

To explore the structure of the environmental data and its relationship with CVD emergency visits, we projected the high‐dimensional environmental features into a two‐dimensional UMAP space. Figure 2 presents the UMAPs generated using the averaged environmental features across Taiwan. Consistent with strong seasonal environmental variability, the UMAPs exhibit clear clustering patterns by month and season, which are directly linked to environmental variations. Specifically, samples from summer are primarily grouped in the upper region, whereas those from winter cluster toward the lower region (Figure 2a). Year‐to‐year variations also influence the UMAP distribution, although their effect is less pronounced than that of seasonality. Regarding CVD emergency‐visit rates (Figure 2b), distinct patterns are observed only in the group aged ≥65 years, where high CVD rates tend to concentrate in the lower‐left corner of the UMAP, forming a visually recognizable cluster.

**Figure 3:**
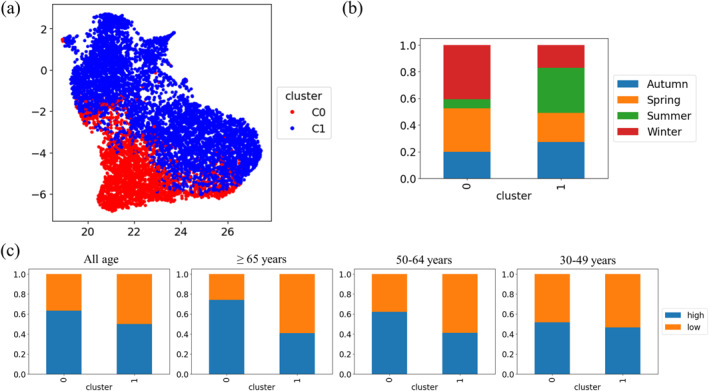
K‐means clustering of daily samples based on environmental features across Taiwan. (a) UMAP visualization showing two clusters (C0 and C1) obtained from K‐means clustering (*k* = 2). (b) Seasonal composition of each cluster. (c) Proportions of high‐ and low‐risk CVD days in clusters C0 and C1 for all individuals, ≥65 years, 50–64 years, and 30–49 years (from left to right).

To quantify these patterns, we applied K‐means clustering (*k* = 2) to partition daily samples in an unsupervised manner based on environmental features. The clustering results are shown in Figure 3a, where two clusters, Cluster 0 (C0) and Cluster 1 (C1), were identified. C0 primarily occupies the lower‐left region of the UMAP, whereas C1 comprises the remaining samples. Both clusters contain samples from all four seasons, though their seasonal compositions differ. Cluster 0 includes relatively fewer summer and autumn samples, while Cluster 1 includes relatively fewer winter and spring samples (Figure 3b). Figure 3c compares the proportions of high‐ and low‐risk CVD days in each cluster across age groups. High risk was defined as CVD emergency visit rate above the group‐specific mean. Across all age groups, C0 contains a significantly higher proportion of high‐risk samples than C1 (see Table 1 for chi‐square test results). In particular, the group aged 65 years and above shows the largest difference (74.2% vs. 40.9%), with the highest chi‐square statistic of 797.3 among all age groups. The second largest difference is observed in the group aged 50–64 years (*χ* ^2^ = 319.0), whereas the group aged 30–49 years shows the smallest difference (*χ* ^2^ = 17.9). These findings suggest that environmental factors may exert a stronger influence on older populations, who are more vulnerable to abrupt environmental changes.

**Table 1:** Comparison of High‐Risk Proportions and Chi‐Square Statistics Between Clusters (C0 and C1) Across Different Populations and Feature Sets for the Whole Taiwan Region

Exp. settings	Age group	Ratio of high CVD risk (C0)	Ratio of high CVD risk (C1)	*χ*2	*p*‐value
All	All	0.634	0.500	128.1	1.09E−29**
	65+	0.742	0.409	797.3	2.07E−175**
	50–64	0.623	0.412	319.0	2.40E−71**
	30–49	0.517	0.467	17.9	2.33E−05**
Female	All	0.673	0.507	200.1	2.01E−45**
	65+	0.713	0.394	727.4	3.27E−160**
	50–64	0.604	0.345	488.0	3.86E−108**
	30–49	0.535	0.406	120.3	5.44E−28**
Male	All	0.548	0.452	32.2	1.41E−08**
	65+	0.702	0.417	581.3	1.94E−128**
	50–64	0.602	0.431	210.7	9.58E−48**
	30–49	0.458	0.468	0.7	0.3942
All (air‐pollution features)	All	0.613	0.507	83.3	7.05E−20**
	65+	0.750	0.398	910.3	5.66E−200**
	50–64	0.634	0.402	394.0	1.10E−87**
	30–49	0.506	0.472	9.0	0.0028**
All (meteorological features)	All	0.638	0.509	105.2	1.13E−24**
	65+	0.638	0.470	179.6	5.98E−41**
	50–64	0.574	0.445	106.1	7.09E−25**
	30–49	0.538	0.464	33.7	6.52E−09**

*Note.* ** indicates statistical significance at *p* < 0.01 (based on the chi‐square test comparing proportions between C0 and C1).

**Figure 4:**
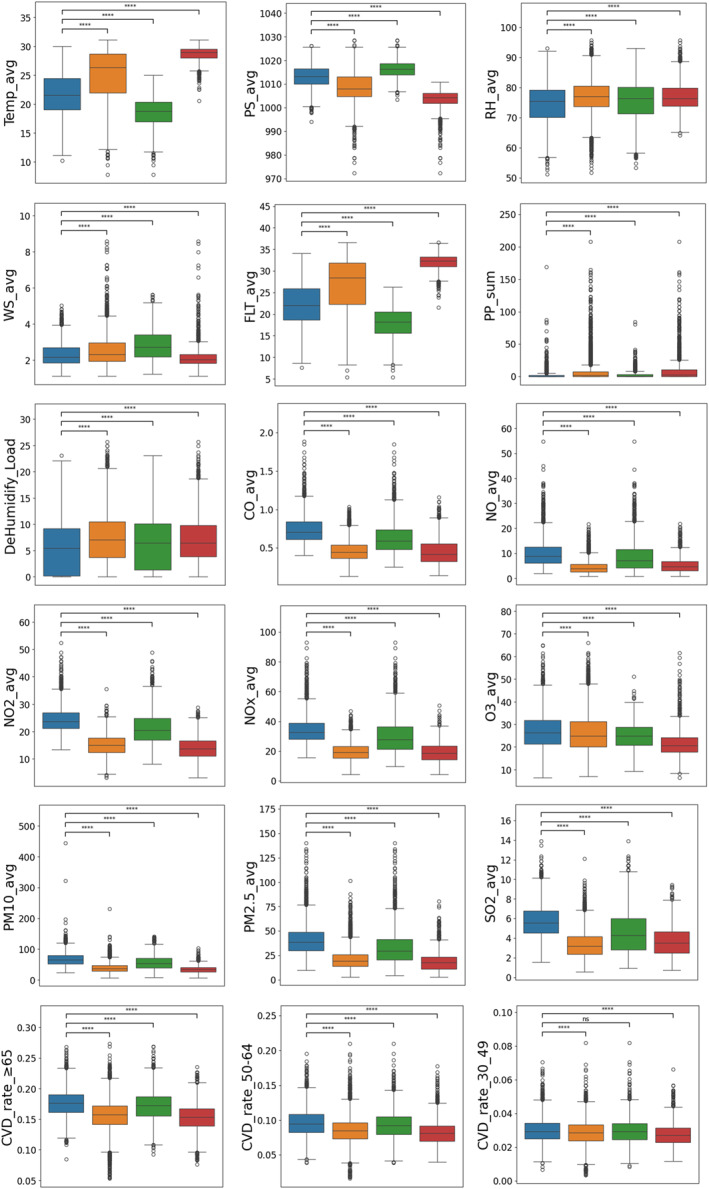
Distributions of daily environmental features and CVD rates across age groups (65+, 50–64, 30–49) in cluster 0 (blue), cluster 1 (orange), winter (green), and summer (red). Environmental features include temperature (Temp_avg), pressure (PS_avg), relative humidity (RH_avg), wind speed (WS_avg), feel‐like temperature (FLT_avg), total rainfall (PP_sum), dehumidification load (DeHumidify_Load; defined as deviation above a 70% relative humidity threshold), and air pollutants, including CO (CO_avg), NO (NO_avg), NO_2_ (NO2_avg), NO_x_ (NO_x__avg), O_3_ (O3_avg), PM_10_ (PM_10__avg), PM_2.5_ (PM_2.5__avg), and SO_2_ (SO2_avg). Statistical significance was assessed using the Mann–Whitney *U* test. Significance levels are denoted as: *p* < 0.05 (*), *p* < 0.01 (**), *p* < 0.001 (***), *p* < 0.0001 (****), and “ns” for non‐significant results. Suffixes “_avg” and “_sum” denote daily average and daily total values, respectively.

To further characterize the distributions of environmental features and CVD rates across clusters, we examined the daily averaged environmental variables and CVD rates in C0, C1, and, for comparison, samples occurring in winter and summer (Figure 4). Overall, C0 and C1 exhibit distinct environmental profiles that also differ from those of winter and summer. For meteorological variables (Temp, PS, RH, WS, FLT, PP), values in both C0 and C1 typically fall between those observed in winter and summer. As expected, C0 more closely resembles winter conditions, whereas C1 is more similar to summer, reflecting the seasonal compositions of the two clusters. In contrast, the air‐pollution features show a markedly different pattern. Across all pollutants (PM_2.5_, PM_10_, O_3_, SO_2_, CO, NO, NO_2_, and NO_x_), C0 consistently exhibits substantially higher concentrations than both C1 and the seasonal groups, indicating that C0 largely captures the most polluted days. Notably, although C0 includes days from all four seasons, the CVD rates among adults aged 65+ and 50–64 remain significantly higher than the rates observed during winter days. These findings together suggest that air‐pollution factors may be more strongly associated with elevated CVD risk than meteorological conditions or season, particularly among older populations.

To evaluate gender‐specific effects, we compared UMAP visualizations of CVD risk between female and male populations separately (see Figures S3 and S4 in Supporting Information S1). The spatial patterns observed in both groups were similar to those of the overall population, with significantly higher CVD risk in cluster C0, particularly among the older age groups (≥65 years and 50–64 years). Notably, the difference in high‐risk ratios was larger in the female population than in the male population (see Table 1). Specifically, the chi‐square statistics were higher in females than in males (female ≥65 years: 727.4; female 50–64 years: 488.0; male ≥65 years: 581.3; male 50–64 years: 210.7), suggesting that CVD risk among females may be more strongly associated with environmental factors.

To isolate the relative contributions of air pollution and meteorology, we repeated the same unsupervised analysis using only one type of environmental feature at a time. The UMAPs generated using air‐pollution and meteorological features separately are shown in Figures S5 and S7 in Supporting Information S1, and the corresponding K‐means clustering results are shown in Figures S6 and S8 in Supporting Information S1. Overall, the patterns remain consistent with those obtained using all environmental features combined. Both air‐pollution and meteorological variables show strong associations with month and season and weaker associations with year‐to‐year variation. Both features also exhibit similar age‐related trends, with stronger clustering patterns in senior groups, as seen in the UMAP visualizations. In the K‐means clustering results, the C0 clusters derived from both feature types show significantly higher CVD risk than the C1 clusters (see detailed chi‐square test results in Table 1). However, the difference in high‐risk ratios between C0 and C1 is much larger when using air‐pollution features compared with meteorological features (air‐pollution ≥65 years: 910.3; air‐pollution 50–64 years: 394.0; meteorological ≥65 years: 179.6; meteorological 50–64 years: 106.1). This strong contrast indicates that air‐pollution factors play a more dominant role in the environmental modulation of CVD emergency visit rates, especially in older adults.

### 3.2 Regional Environmental Factor Analysis Using Unsupervised Learning Methods

**Figure 5:**
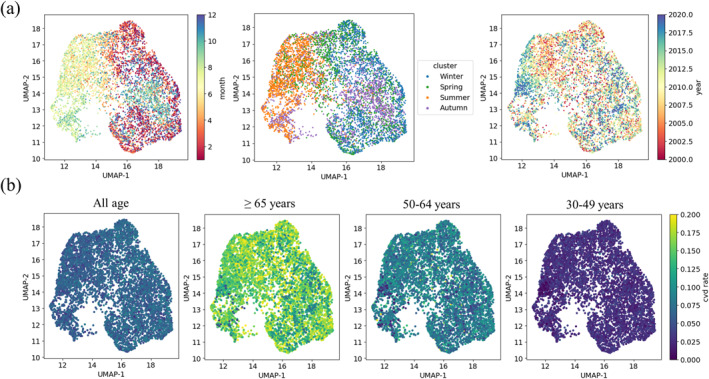
UMAP visualization of environmental features and CVD emergency‐visit rates in the TNKY region. (a) UMAP projections of averaged environmental features in the TNKY region, colored by month, season, and year (from left to right). (b) UMAPs colored by CVD emergency‐visit rates for all individuals, ≥65 years, 50–64 years, and 30–49 years (from left to right).

Following the previous section, we further conducted unsupervised learning analyses for each of the five Taiwan regions individually. Figure 5 presents the results for the TNKY region, while the other four regions are shown in Figure S9 (THM), Figure S11 (YCTKP), Figure S13 (TCN), and Figure S15 (HT) in Supporting Information S1. Across all regions, UMAP visualizations indicate that environmental features remain strongly organized by month and season, consistent with the nationwide pattern. Among the five regions, TNKY exhibits the clearest association between environmental conditions and CVD emergency visit rates in the senior population. In contrast, such patterns were less visually distinct in the other regions.

**Figure 6:**
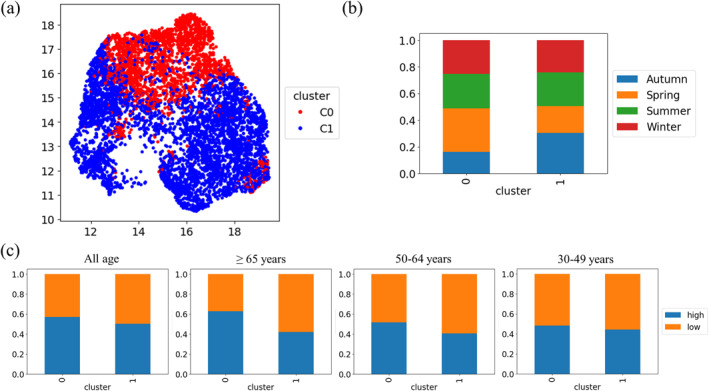
K‐means clustering of daily samples based on environmental features in the TNKY region. (a) UMAP visualization showing two clusters (C0 and C1) obtained from K‐means clustering (*k* = 2). (b) Seasonal composition of each cluster. (c) Proportions of high‐ and low‐risk CVD days in clusters C0 and C1 for all individuals, ≥65 years, 50–64 years, and 30–49 years (from left to right).

The K‐means clustering results for the TNKY region are shown in Figure 6, and those for the remaining regions are shown in Figure S10 (THM), Figure S12 (YCTKP), Figure S14 (TCN), and Figure S16 (HT) in Supporting Information S1. In the TNKY region, cluster C0 corresponds to the top‐middle area of the UMAP, whereas the remaining samples form cluster C1. Similar to the clustering result for the whole Taiwan region, both C0 and C1 contain samples from all four seasons, and the seasonal distribution is more even in this region. C0 includes relatively fewer autumn samples, and C1 includes relatively fewer spring samples. These patterns suggest that the clustering result may reflect a more complex interaction among multiple environmental features rather than a single dominant seasonal effect. C0 generally exhibited higher CVD risk than C1, particularly for the group aged ≥65 years (see Table 2 for details). This difference was also observed in other regions, except for HT, likely due to its smaller population size (see Table 2, where the p‐values for the ≥65 group are below 0.05 in TNKY, THM, YCTKP, and TCN). Considering the chi‐square statistics, only the ≥65 group in TNKY showed a value exceeding 200, while those in other regions were substantially lower (typically below 100). These results suggest that the influence of environmental factors on CVD risk is strongest in the TNKY region, which also has the largest population among the five regions.

**Table 2:** Comparison of High‐Risk Proportions and Chi‐Square Statistics Between Clusters (C0 and C1) Across Different Populations in Five Different Regions

Region	Age group	Ratio of high CVD risk (C0)	Ratio of high CVD risk (C1)	*χ*2	*p*‐value
TNKY	All	0.572	0.504	27.0	1.98E−07**
	65+	0.628	0.421	248.9	4.43E−56**
	50–64	0.516	0.406	72.3	1.87E−17**
	30–49	0.483	0.443	9.7	0.0019**
THM	All	0.490	0.520	2.9	0.0868
	65+	0.622	0.472	75.6	3.54E−18**
	50–64	0.502	0.481	1.4	0.2413
	30–49	0.407	0.482	18.9	1.37E−05**
YCTKP	All	0.574	0.450	102.6	4.10E−24**
	65+	0.517	0.458	24.3	8.30E−07**
	50–64	0.495	0.435	24.4	8.03E−07**
	30–49	0.494	0.437	22.1	2.58E−06**
TCN	All	0.598	0.483	59.8	1.06E−14**
	65+	0.509	0.465	8.7	0.0031**
	50–64	0.454	0.480	3.0	0.0817
	30–49	0.472	0.460	0.6	0.4450
HT	All	0.420	0.515	11.3	0.0008**
	65+	0.503	0.455	2.9	0.0884
	50–64	0.425	0.491	5.4	0.0204*
	30–49	0.437	0.404	1.4	0.2400

*Note.* * and ** indicate statistical significance at *p* < 0.05 and *p* < 0.01, respectively (based on the chi‐square test comparing proportions between C0 and C1).

### 3.3 Prediction Performance Across Supervised Learning Models

**Figure 7:**
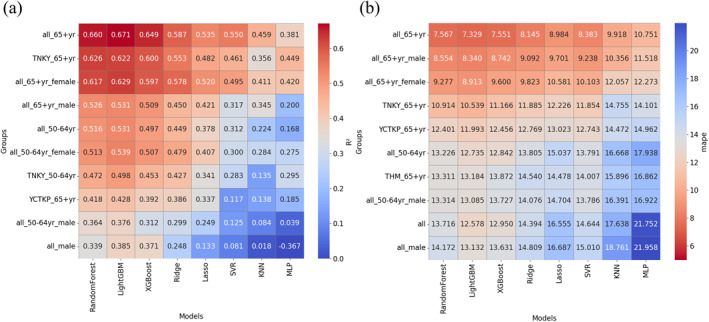
Performance of supervised machine‐learning models for predicting daily CVD emergency‐visit rates in the top ten demographic–regional groups with the best overall predictability. (a) Heatmap of model performance quantified by the mean *R* ^2^ between predicted and observed daily CVD emergency visits during 2000–2022. (b) Heatmap of model performance quantified by the Mean Absolute Percentage Error (MAPE). For both panels, warmer colors indicate better predictive performance (higher *R* ^2^ or lower MAPE), whereas cooler colors indicate poorer performance. Each cell represents the average performance for a given model and population group.

Figure 7a compares the predictive performance of eight machine‐learning algorithms for the top ten demographic–regional groups (see Figure S17 in Supporting Information S1 for results across all groups), expressed as *R* ^2^. The three tree‐based models achieved the highest *R* ^2^ values, reaching 0.65–0.67, whereas all non–tree‐based models remained below 0.60. The tree‐based models exhibited consistently higher and more uniform *R* ^2^ values across population subsets, highlighting their robustness to data normalization and feature scaling.

Among the tree‐based models (Random Forest, LightGBM, and XGBoost), predictive performance was broadly comparable, with *R* ^2^ values typically ranging between 0.60 and 0.67 for the nationwide elderly group and slightly lower for regional subsets. The northern region (TNKY in Figure 1) exhibited the strongest regional predictability (*R* ^2^ ≈ 0.60–0.63 for the ≥65‐year group). This enhanced performance likely reflects denser population coverage and higher‐quality environmental monitoring data in northern Taiwan, which reduce data noise and increase model stability.

In contrast, the non–tree‐based models (Ridge, LASSO, SVR, KNN, and MLP) produced systematically lower *R* ^2^ values, ranging from 0.36 to 0.56 in the elderly group. Ridge regression achieved the best performance among these models (*R* ^2^ = 0.55 for TNKY_65+), followed by LASSO (*R* ^2^ = 0.48), SVR (*R* ^2^ = 0.46), and MLP (*R* ^2^ = 0.45). The KNN model yielded the lowest *R* ^2^, consistent with the difficulty of computing meaningful distances in high‐dimensional environmental feature spaces. The overall reduction in performance relative to tree‐based models reflects the limited capacity of these algorithms to capture nonlinear dependencies and multivariate interactions among environmental predictors.

Across all supervised models, predictive power was highest for elderly populations (≥65 years), followed by the 50–64‐year and 30–49‐year groups. This trend was consistent across model types, suggesting that older adults exhibit stronger and more predictable associations between environmental variability and daily CVD emergency visits. Notably, elderly females (≥65 years) showed slightly higher *R* ^2^ values than males of the same age group. Regionally, northern Taiwan exhibited more stable and robust predictive relationships, likely due to denser station coverage and more consistent air‐pollution regimes. Together, these trends confirm that environmental predictability of CVD emergency visit is strongest for elderly populations in northern Taiwan and that ensemble tree algorithms provide the most effective modeling framework.

In addition to *R* ^2^, model performance can be also evaluated using MAPE, to assess absolute prediction accuracy (Figure 7b shows the top ten demographic–regional groups; see Figure S18 in Supporting Information S1 for all groups). Across all population subsets, tree‐based models again demonstrated superior performance, achieving the lowest MAPE values (approximately 7%–11%) for both national and regional elderly groups. Random Forest, LightGBM, and XGBoost produced nearly identical MAPE ranges, consistent with their comparable *R* ^2^ results, indicating that these ensemble methods not only captured temporal variability but also maintained small absolute prediction errors.

By comparison, the non–tree‐based models (Ridge, LASSO, SVR, KNN, and MLP) yielded slightly higher MAPE values (approximately 8%–15%), consistent with their relatively lower *R* ^2^ values. Their reduced flexibility in modeling nonlinear relationships with interacting atmospheric processes likely limits their ability to track short‐term fluctuations in cardiovascular risk.

Overall, the *R* ^2^ and MAPE results consistently demonstrate that ensemble tree models achieve the best balance between capturing variability and maintaining predictive stability. Owing to their high *R* ^2^, low MAPE, and proven robustness to feature scaling, the tree‐based algorithms were selected for subsequent SHAP analysis to identify key environmental drivers influencing CVD emergency risk.

### 3.4 Interpretability via SHAP

**Figure 8:**
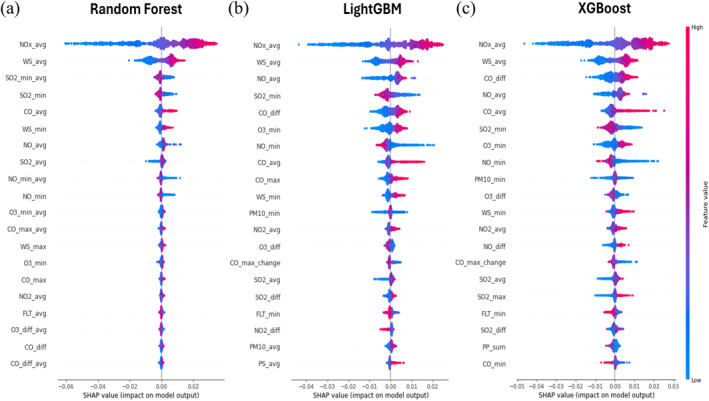
Comparison of feature importance ranked by SHAP values for the three tree‐based models: (a) Random Forest, (b) LightGBM, and (c) XGBoost. Each point represents the SHAP value for an individual prediction, where color indicates the feature value (blue for low, red for high). Features are ordered by their mean absolute SHAP value across all samples.

Figure 8 summarizes feature importance rankings derived from SHAP values for the three ensemble tree models: (a) Random Forest, (b) LightGBM, and (c) XGBoost. Each point represents the SHAP value for an individual prediction, with color indicating the feature value (blue for low, red for high). Features are ordered by their mean absolute SHAP value across all samples providing a consistent measure of their overall contribution to model predictions.

Across all three models, NO_x____avg consistently exhibits the strongest influence on predicted CVD emergency visits, followed by gaseous pollutants such as WS_avg, NO_avg, CO_diff, SO_2____min, and O_3____min. These features collectively capture both air quality variability and atmospheric dispersion conditions that modulate pollutant accumulation or dispersion, indicating that these environmental processes play a dominant role in shaping short‐term cardiovascular risk.

Although the three ensemble architectures differ in how they partition feature space and handle correlated variables, the overall feature importance patterns are remarkably consistent. Minor differences among models likely reflect variations in how each algorithm represents nonlinear interactions or redundant predictors, but the core result is robust: gaseous pollutants particularly NO_x_ and related nitrogen species, exert the strongest and most consistent influence on predicted CVD emergency visit rates across Taiwan.

## 4 Discussion

Our findings demonstrate that air‐pollution factors, rather than meteorological conditions alone, are the primary environmental drivers contributing to cardiovascular events among older populations, a pattern consistently observed across both supervised and unsupervised learning analyses. Among these factors, NO_x_ consistently emerged as the most influential predictor of cardiovascular emergency visits, particularly in the urban centers of northern Taiwan. This observation aligns with growing global evidence linking NO_x_ exposure to elevated risks of CVD morbidity and mortality in densely populated regions. The higher predictive accuracy observed in the supervised learning models and the distinct high‐risk cluster revealed by the unsupervised analyses in metropolitan areas, compared with more industrialized or rural regions, likely reflect both the unique chemical composition of urban air pollution and the structural characteristics of city environments that amplify exposure and vulnerability.

### 4.1 Machine‐Learning Approaches for Characterizing Environmental Impacts on CVD

Our findings demonstrate how machine‐learning methods can extract structure from environmental–health data that would be difficult to detect using conventional linear or single‐pollutant approaches. The unsupervised analyses show that nonlinear manifold learning (UMAP) and clustering (K‐means) can organize thousands of daily environmental profiles into a small number of data‐driven “regimes” that exhibit patterns distinct from traditional seasonal categories and that are associated with elevated CVD risk among older adults. This provides an integrated view of exposure patterns, revealing how combinations of temperature, humidity, and multiple pollutants jointly define high‐risk states, rather than focusing on isolated variables or pre‐defined thresholds. In addition, regional analyses highlight that these data‐driven regimes differ across Taiwan's climatic zones, offering a flexible framework to explore spatial heterogeneity in environmental sensitivity.

In the supervised setting, ensemble tree models capture complex nonlinear effects and interactions among meteorological and air‐pollution predictors, enabling quantitative prediction of daily CVD emergency‐visit rates at both national and regional scales. Compared with traditional regression, these models can accommodate high‐dimensional, correlated features without extensive manual selection or transformation, while SHAP‐based interpretation translates their “black‐box” predictions into ranked, directionally interpretable drivers. The consistent prominence of NO_x_‐related and gaseous‐pollution metrics across models illustrates how machine learning can identify key exposure patterns that emerge from the joint distribution of many variables, guiding hypothesis generation and informing targeted environmental interventions. Collectively, this framework shows that modern machine‐learning tools can complement conventional methods by uncovering multivariate risk regimes, enhancing short‐term predictability, and providing interpretable summaries of complex exposure–response relationships.

### 4.2 Why NO_x_ Is a Key Environmental Predictor of CVD

NO_x_ stands out as a dominant predictor primarily because of its urban source dominance, acute cardiovascular effects, and spatial specificity. First, NO_x_ is mainly emitted from vehicle exhaust and other combustion‐related urban sources, making it a direct indicator of traffic‐related air pollution (de Bont et al., 2022; Niepsch et al., 2022; Prathibha et al., 2023). In highly populated cities with dense traffic networks, NO_x_ concentrations vary sharply over small spatial scales, providing a high‐resolution signal that captures day‐to‐day exposure changes relevant to cardiovascular risk. Second, the acute cardiovascular toxicity of NO_x_ is well established. Numerous time‐series studies report that short‐term increases in NO_x_ or NO_2_ concentrations are significantly associated with elevated risks of hypertension, myocardial infarction, arrhythmia, and stroke (Czernych et al., 2023; Luo et al., 2016). These effects are often more pronounced than those of PM_2.5_ or O_3_ in urban contexts, suggesting that NO_x_ acts as both a surrogate for local traffic emissions and a direct agent of oxidative stress and endothelial dysfunction. Finally, the spatial specificity of NO_x_ exposure enhances its predictive power. Unlike PM_2.5_, which tends to exhibit greater spatial homogeneity at the regional scale relative to NO_x_, NO_x_ varies substantially across neighborhoods depending on road density, traffic flow, and street canyon geometry. This localized variability allows NO_x_‐based models to better capture the spatial and temporal dynamics of CVD triggers in urban settings.

### 4.3 Why Big Cities Outperform Industrial Cities in Model Prediction

The stronger predictive performance and distinct clustering pattern observed in northern Taiwan's metropolitan region (TNKY) can be explained by three interrelated factors: population density and data quality, pollution source characteristics, and urban form and healthcare access.

First, high population density, extensive medical and monitoring infrastructure provide high‐quality, temporally consistent health and exposure data, reducing noise and improving model stability (Shen & Lung, 2020).

Second, pollution sources in large cities are more uniform, with traffic emissions dominating the pollutant mixture. This results in NO_x_ being more representative of urban pollutant conditions, making it more consistent and interpretable indicator of short‐term pollution stress (Carlsen et al., 2022; de Bont et al., 2022). In contrast, central and southern Taiwan are more strongly influenced by industrial emissions and complex regional transport processes, leading to a more heterogeneous mixture of pollutants, including elevated contributions from PM_2.5_ and other secondary aerosols (S.‐L. Lin et al., 2022; J.‐H. Tsai et al., 2024; Wu & Huang, 2021). This increased compositional complexity may reduce the ability of single indicators to capture short‐term variability in exposure, thereby weakening predictive performance.

Third, urban form and health accessibility influence both exposure and response. Mixed land use, dense transportation networks, and advanced medical infrastructure in metropolitan areas create conditions in which pollutant exposure and healthcare utilization are both more measurable and more directly coupled to acute outcomes. These structural advantages lead to stronger observed relationships between NO_x_ fluctuations and cardiovascular emergency visits.

### 4.4 Sex‐Specific Susceptibility to Environmental Cardiovascular Risk Factors

Our findings indicate that elderly females exhibited slightly stronger associations between environmental exposures and CVD outcomes than males of the same age group, as reflected by higher chi‐square statistics (Table 1) and *R* ^2^ values (Figure 7a). This observation is consistent with several epidemiological studies reporting sex‐specific vulnerability to air pollution. For instance, studies stratified by sex have shown that females often experience higher relative risks of cardiovascular outcomes associated with PM_2.5_ and PM_10_ exposure, suggesting greater susceptibility to particulate pollution (F. Lin et al., 2025; Liu et al., 2020; Zhang et al., 2022).

These differences may arise from a complex interplay of physiological and hormonal factors affecting vascular reactivity, as well as sociobehavioral influences such as exposure patterns and healthcare‐seeking behavior. However, the available evidence remains limited, and the underlying mechanisms are not fully understood (Zhou et al., 2025). Our results suggest that environmental sensitivity may differ by sex, particularly in older populations, although further mechanistic studies are required to clarify the basis of these differences.

### 4.5 Model Limitation, Future Works, and Implications

Using 23 years of daily data (2000–2022), this study constructed predictive models linking environmental variability to cardiovascular emergency visits across Taiwan. Among all models, the tree‐based models achieved the best performance, with nationwide elderly groups (≥65 years) showing *R* ^2^ values around 0.60–0.67 and minimum MAPE values of 7%–8%. These results indicate that although the models capture meaningful variability, their predictive accuracy remains moderate, which highlights key methodological limitations and opportunities for further improvement.

The fact that *R* ^2^ values did not exceed 0.7 suggests that a substantial proportion of daily CVD variability remains unexplained. In addition, the best‐performing tree‐based models yielded MAPE around 7%–8%, indicating modest absolute accuracy for day‐to‐day forecasting. While this level of precision is acceptable for identifying environmental sensitivity, it remains insufficient for operational early‐warning applications that require finer resolution or probabilistic thresholds. Linear models occasionally produced similar or slightly lower MAPE despite lower *R* ^2^, reflecting their conservative predictions that regress toward the mean—stable in magnitude but poor in temporal tracking. This reinforces the use of tree‐based models for interpretability and dynamic response analysis.

Another limitation of this study is that default hyperparameters were applied uniformly across all supervised learning models. Because some algorithms (e.g., KNN and SVR) are particularly sensitive to hyperparameter choices in high‐dimensional settings, the use of default configurations may have disadvantaged certain models and affected their relative performance. Future studies could incorporate systematic hyperparameter optimization to further evaluate the robustness and comparative performance of different algorithms. For the unsupervised learning component, *k* = 2 was chosen to distinguish high‐ and low‐risk groups. Nevertheless, the selection of *k* may be adapted based on specific analytical objectives (e.g., increasing *k* to capture more fine‐grained subgroup structures) or determined using data‐driven methods such as the elbow method.

It is also worth noting that emergency visits are influenced not only by environmental triggers but also by other factors such as underlying disease burden, healthcare accessibility, holidays, influenza outbreaks, and policy changes (e.g., during COVID‐19). These unmeasured influences dilute the environmental signal and limit explainable variance. Exposure uncertainty can also introduce uncertainty: Station‐based daily averages are used as population‐level exposure proxies, introducing spatial misalignment and indoor–outdoor differences. For pollutants like NO_x_, which show steep spatial gradients near traffic corridors, exposure misclassification constrains achievable model fit. Furthermore, the NO_x_, NO, and NO_2_ features included in the supervised models are highly intercorrelated; SHAP values may be distributed unpredictably across these collinear predictors, potentially understating the aggregate importance of the nitrogen oxide family. Another error source could be associated with temporal structure. The models use daily averages and do not fully incorporate lagged or extreme‐event dynamics (e.g., cold surges, heatwaves, or abrupt humidity changes). Ignoring such temporal complexity likely underestimates transient cardiovascular responses.

Despite these limitations, our findings underscore the dual role of NO_x_ as both a sensitive tracer of urban air pollution and a physiologically relevant driver of acute cardiovascular events. The consistently strong predictive performance in traffic‐dense cities highlights the need for fine‐scale air quality monitoring and targeted mitigation strategies focused on NO_x_ reduction in metropolitan regions.

## 5 Conclusions

Using 23 years of nationwide data from Taiwan, this study shows that high‐dimensional meteorological and air‐pollution features can be organized into distinct environmental regimes that differ from conventional seasonal categories and, importantly, correspond to elevated CVD emergency‐visit rates among older adults. Unsupervised learning reveals that specific environmental states (cluster C0) consistently contain higher proportions of high‐risk CVD days, especially among individuals aged 65 years and older and, to a lesser extent, those aged 50–64 years, with stronger associations in females than males. Air‐pollution variables produce larger contrasts in high‐risk proportions than meteorological variables alone, indicating that pollution variability plays a particularly important role in triggering acute cardiovascular events.

Regional analyses further show that these patterns are strongest in northern Taiwan (TNKY), where denser population and monitoring coverage support clearer links between environmental regimes and CVD risk. Supervised machine‐learning models confirm and quantify these relationships. Ensemble tree‐based models (Random Forest, LightGBM, XGBoost) outperform linear and kernel‐based approaches, achieving *R* ^2^ values of about 0.65–0.67 and MAPE of approximately 7%–11% for elderly groups, especially in northern Taiwan, demonstrating that nonlinear, multivariate environmental signals can provide useful short‐term prediction of CVD emergencies. SHAP analyses consistently highlight NO_x_‐related metrics and other gaseous pollutants, together with wind‐related variables, as key predictors, underscoring the joint roles of emission intensity and atmospheric dispersion.

Collectively, these findings show that integrating environmental monitoring with machine‐learning methods offers a powerful framework for identifying vulnerable populations, high‐risk environmental regimes, and dominant exposure drivers, thereby supporting more targeted early‐warning and public‐health interventions under evolving climate and air‐quality conditions.

## Conflict of Interest

The authors declare no conflicts of interest relevant to this study.

## Supporting information

Supporting Information S1

## Data Availability

All environmental data used in this analysis were obtained from publicly available sources. Air quality measurements were retrieved from the Taiwan Air Quality Monitoring Network at https://airtw.moenv.gov.tw/ENG/default.aspx (by clicking “Data Download,” where annual regional data are provided). Meteorological data were retrieved from the Central Weather Administration's Climate and Data Interface Service (CODiS) at https://codis.cwa.gov.tw/ (by clicking “Data Browsing” and following the provided instructions). The code for this study is available in the Zenodo repository (chenh2lab, 2026).
