# Supplementary material for: Predicting Daily Cardiovascular Emergencies Using Weather and Air Quality Data: A 23‐Year Machine‐Learning Analysis in Taiwan
Source: Geohealth. 2026 Jun 12;10(6):e2025GH001769. doi: 10.1029/2025GH001769 (PMC13261087; doi:10.1029/2025GH001769)
Supplement: Supplementary file 1 — Supporting Information S1 [file GH2-10-e2025GH001769-s001.docx]

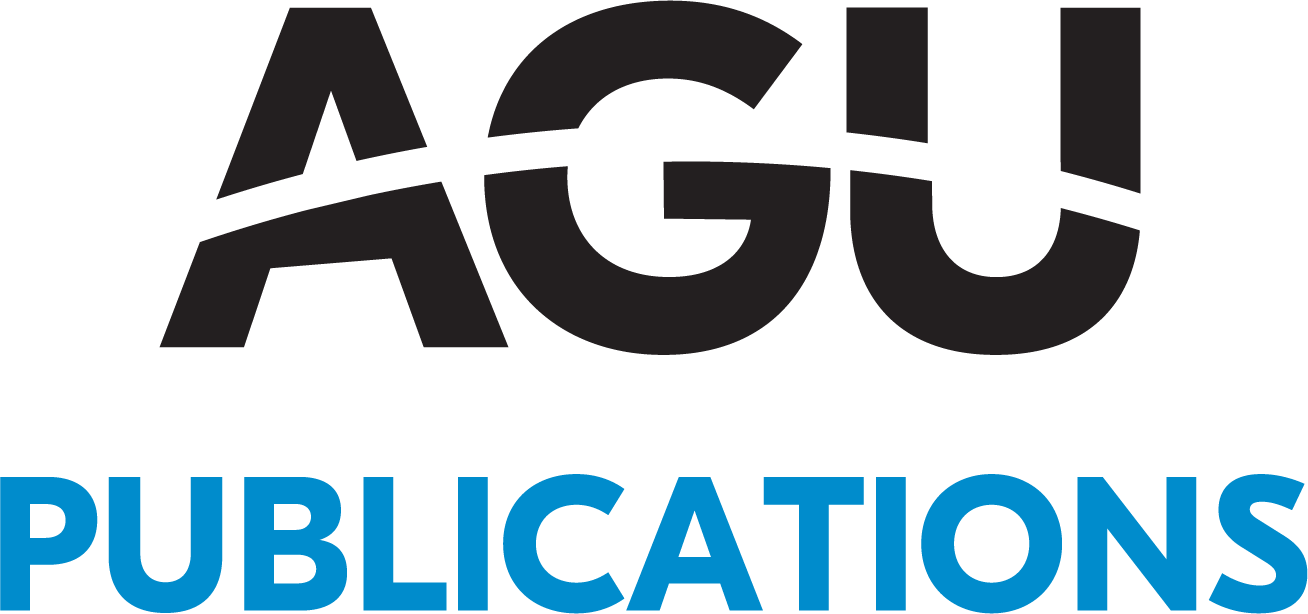


***GeoHealth***

**Supporting Information for**

**Predicting Daily Cardiovascular Emergencies Using Weather and Air Quality Data: A 23-Year Machine-Learning Analysis in Taiwan**

Hsiang-Han Chen^1^, Pei-Shan Tsai^2^, Yu-Chia Chen^1^, Cheng-Yu Li^1^, Yu-Kai Lin^3^, Wan-Ru Huang^2^, Kate Huihsuan Chen^2^

^1^ Department of Computer Science and Information Engineering, National Taiwan Normal University, Taipei, Taiwan.

^2^ Department of Earth Sciences, National Taiwan Normal University, Taipei, Taiwan.

^3^ The Department of Health and Welfare, University of Taipei, Taipei, Taiwan.

Corresponding author: Hsiang-Han Chen ([chenh2@ntnu.edu.tw)](mailto:email@address.edu))

**Contents of this file**

- Text S1 to S2
- Figures S1 to S18

**Introduction**

This supplementary file includes detailed analyses of seasonality in CVD incidence alongside meteorological conditions (Section S1) and air quality (Section S2). It also contains unsupervised learning results for different genders (Figures S3–S4), environmental feature subsets (Figures S5–S6), and individual regions (Figures S7–S16), as well as complete heatmaps summarizing model performance (Figures S17–S18).

**S1. Seasonality of CVD Incidence and Meteorological Condition**

The detrended CVD-related emergency visits exhibit a pronounced **annual cycle** (Figure S1). Across Taiwan, the number of CVD-related emergencies typically peaks during **winter to early spring (December–March)** and reaches a minimum during **summer (June–September)**, consistent with the general pattern of seasonal CVD risk observed in temperate and subtropical climates. **Air pressure (PS)** (Figure S1a) displays a synchronous winter maximum with CVD visits, both reaching their highest values between December and February. **Wind speed (WS)** (Figure S1b) also shows higher values during winter, consistent with the northeasterly monsoon season. These periods of strong wind are typically accompanied by sharp temperature drops, dry air, and elevated CVD rates. Such correspondence indicates that **cold, high-pressure air masses, and wind-related cold exposure may** contribute to conditions that exacerbate cardiovascular stress.

**Precipitation (PP)** (Figure S1c) exhibits an opposite seasonal phase, with most of the rainfall occurring during late spring to early autumn (May–September). **Temperature (Temp)** (Figure S1d) is strongly anti-correlated with CVD emergency counts. As temperature declines during winter, CVD visits rise sharply; conversely, during warm and humid summer months, CVD visits are at their lowest. **Relative humidity (RH)** (Figure S1e) in general, coincide with precipitation, thus reveal similar correlation with PP against CVD incidence. The driest months with low temperature coincide with elevated emergency visits, while the warm–humid, wet season is associated with fewer events.


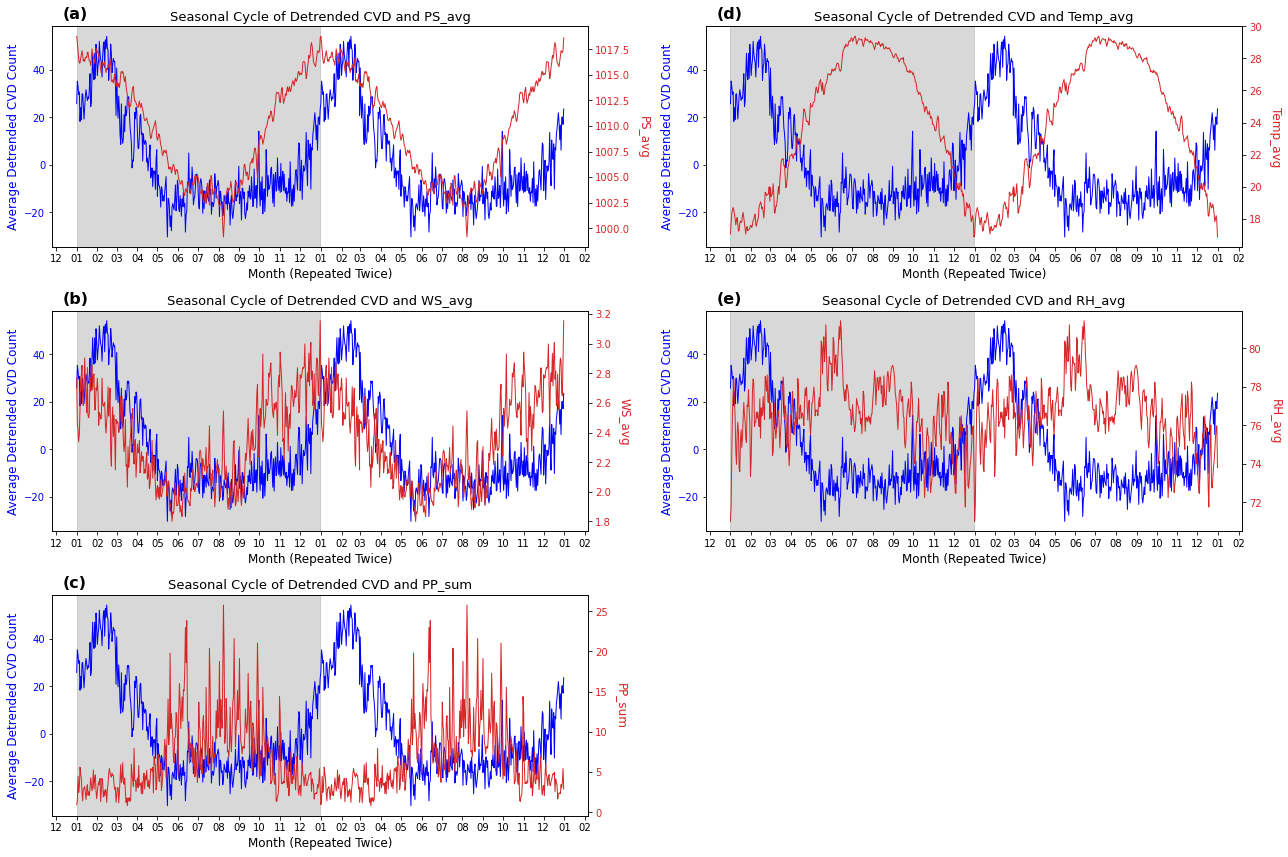


Figure S1. **Seasonal cycles of detrended cardiovascular emergency visits (blue) and major meteorological variables (red) from 2000–2022.** Each panel shows the mean monthly variation of detrended daily data, repeated twice along the x-axis (months 1–12, shown twice) to highlight annual periodicity. The panels display (a) air pressure (PS), (b) wind speed (WD), (c) precipitation (PP), and (d) temperature (Temp), and (e) relative humidity (RH), The shaded area marks the repeated portion of the cycle and does **not** indicate a specific season. Filled triangles indicate the peaks, while open triangles denote the trough.

**S2. Seasonality of CVD Incidence and Air Quality**

Air-pollution variables also exhibit strong and recurrent annual cycles that align closely with the seasonal fluctuations of CVD emergency visits (Figure S2). Most primary pollutants, including **NOₓ (NO₂ and NO reveals highly similar trend with Nox and thus not shown), CO, SO₂, PM₁₀, and PM₂.₅**, reach their **highest concentrations during winter to early spring (December–March)** and decline markedly during **summer to early autumn (June–September)**. This pattern coincides with the seasonal maximum in CVD emergency visits, indicating that **pollution accumulation under winter meteorological conditions** acts as a significant environmental stressor for cardiovascular health.

Both NOₓ and CO exhibit pronounced winter peaks (Figures S2a–b) that mirror the CVD cycle. These pollutants primarily originate from vehicular and combustion sources and tend to accumulate under stable, high-pressure winter conditions with shallow boundary layers. SO₂, PM₁₀, and PM₂.₅ show similar winter maxima (Figures S2d–f), reflecting both industrial and transboundary influences during the northeast monsoon season. These pollutants remain elevated through early spring and decline rapidly after May when rainfall and convective mixing intensify. In contrast, **O₃ displays an opposite seasonal phase** (Figure S2c), peaking in late spring to early summer when photochemical activity is strongest and primary pollutant levels are lower.


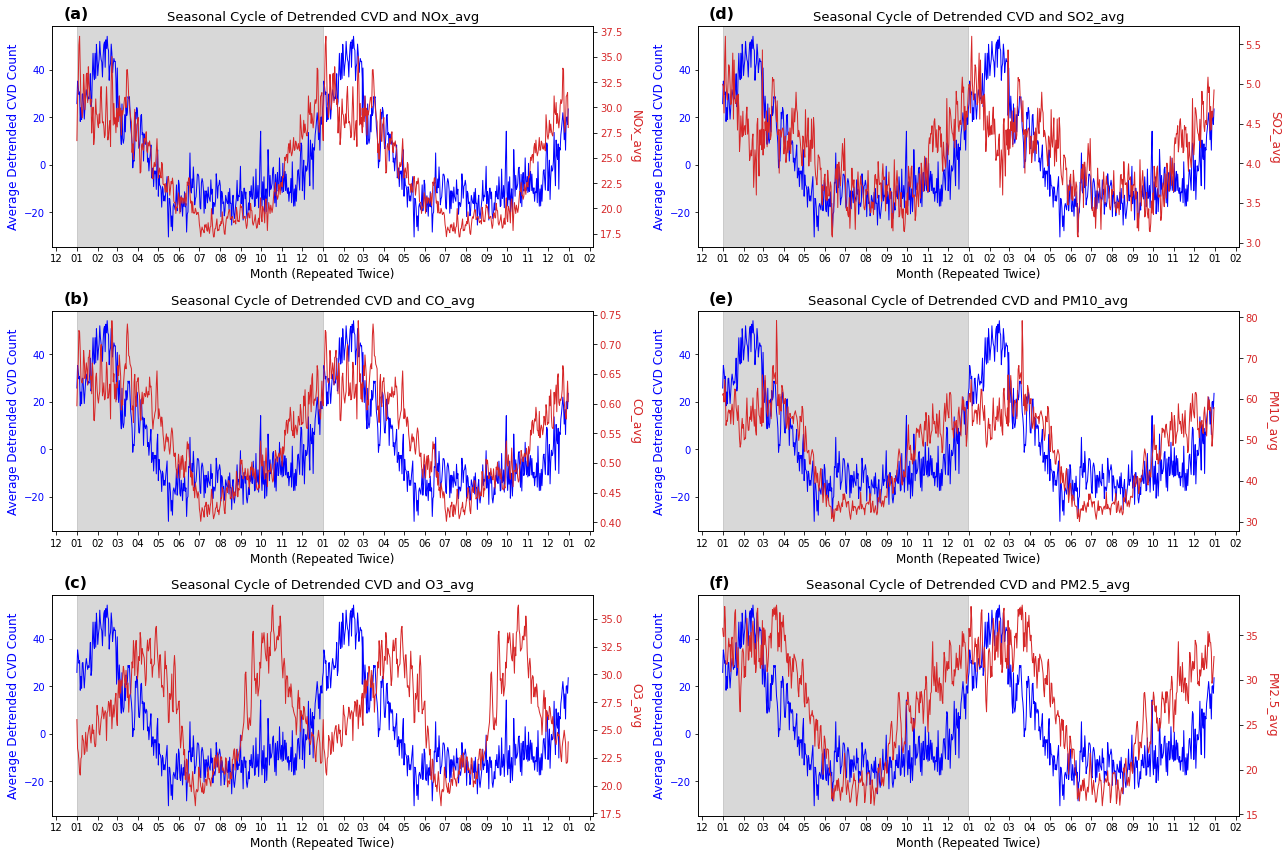


Figure S2. **Seasonal cycles of detrended cardiovascular emergency visits (blue) and air-pollution variables (red) during 2000–2022.** Each panel displays the mean monthly variation of detrended daily data, plotted twice along the x-axis (months 1–12 repeated) to emphasize annual periodicity. The shaded portion represents the repeated section of the cycle and does **not** correspond to a specific season. Panels show (a) nitrogen oxides (NOₓ), (b) carbon monoxide (CO), (c) ozone (O₃), (d) sulfur dioxide (SO₂), (e) particulate matter ≤10 μm (PM₁₀), and (f) particulate matter ≤2.5 μm (PM₂.₅).


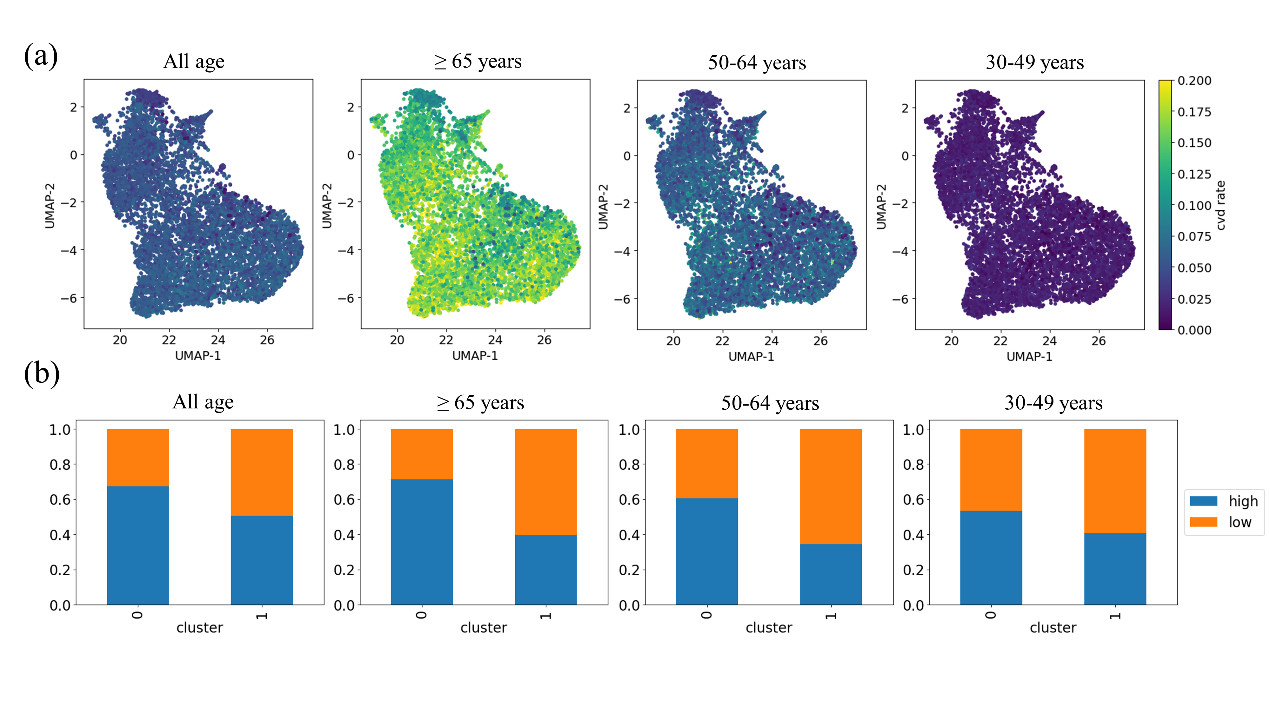
Figure S3. UMAP visualization of environmental features and CVD emergency-visit rates across Taiwan (female population). (a) UMAPs colored by CVD emergency-visit rates for all individuals, ≥65 years, 50–64 years, and 30–49 years (from left to right). (b) Proportions of high- and low-risk CVD days in clusters C0 and C1 for all individuals, ≥65 years, 50–64 years, and 30–49 years (from left to right).


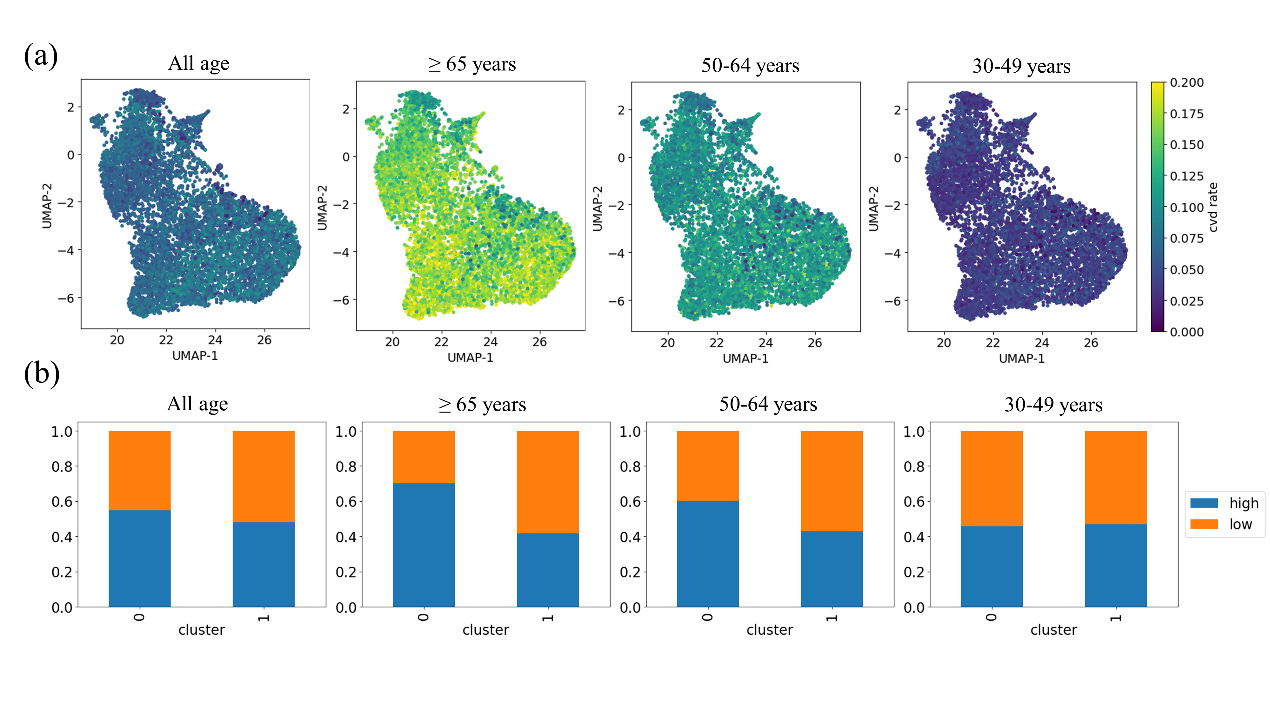


Figure S4. UMAP visualization of environmental features and CVD emergency-visit rates across Taiwan (male population). (a) UMAPs colored by CVD emergency-visit rates for all individuals, ≥65 years, 50–64 years, and 30–49 years (from left to right). (b) Proportions of high- and low-risk CVD days in clusters C0 and C1 for all individuals, ≥65 years, 50–64 years, and 30–49 years (from left to right).


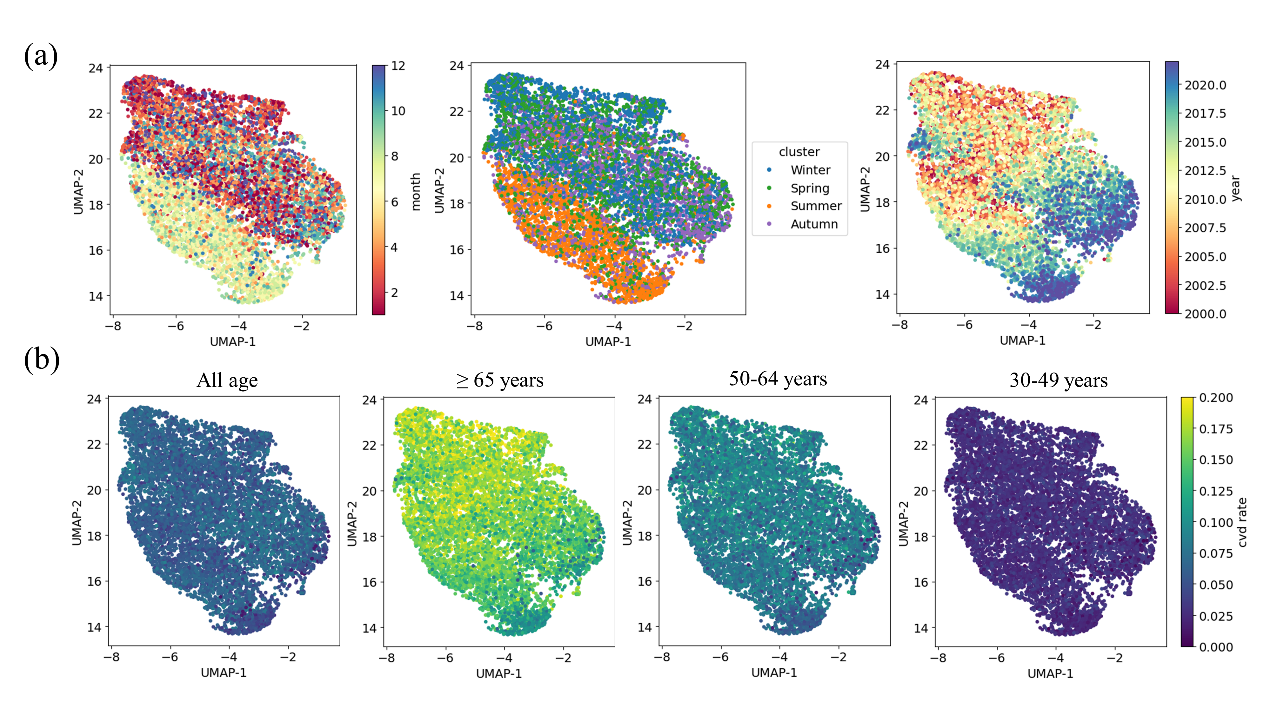


Figure S5. UMAP visualization of environmental features and CVD emergency-visit rates across Taiwan (using air-pollution features). (a) UMAP projections of averaged environmental features across Taiwan, colored by month, season, and year (from left to right). (b) UMAPs colored by CVD emergency-visit rates for all individuals, ≥65 years, 50–64 years, and 30–49 years (from left to right).


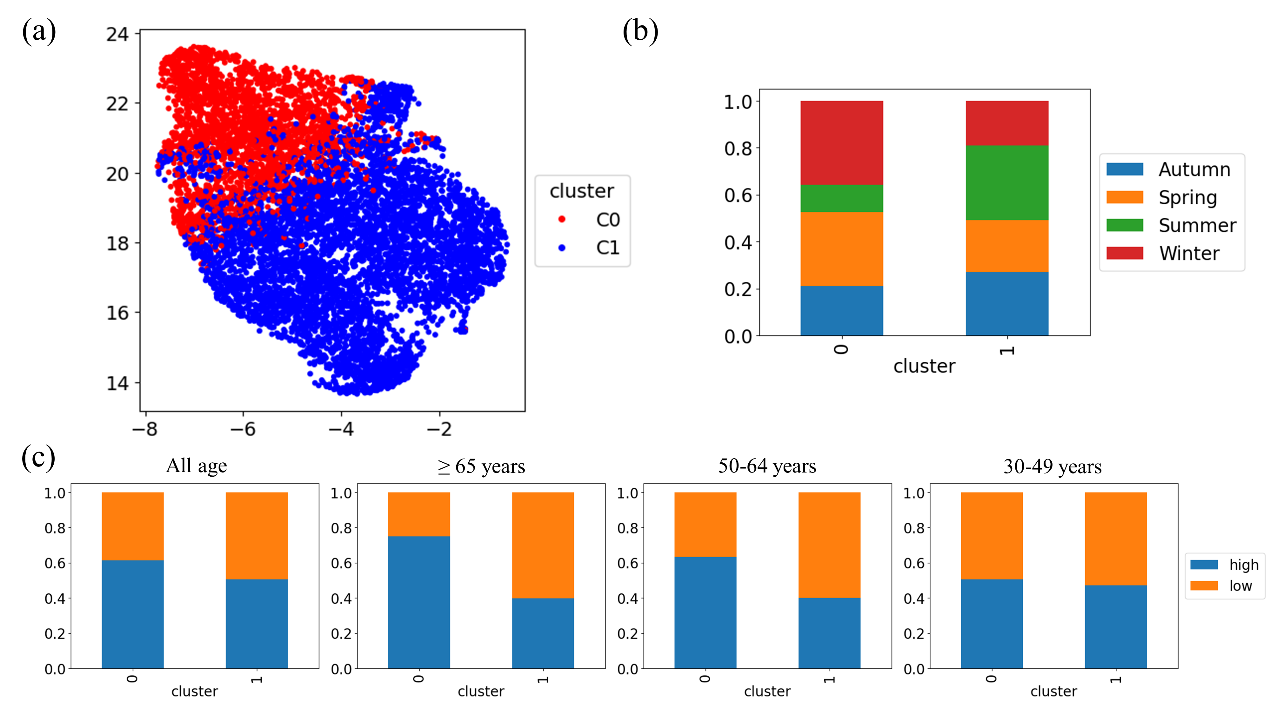
Figure S6. K-means clustering of daily samples based on environmental features across Taiwan (using air quality features). (a) UMAP visualization showing two clusters (C0 and C1) obtained from K-means clustering (k = 2). (b) Seasonal composition of each cluster. (c) Proportions of high- and low-risk CVD days in clusters C0 and C1 for all individuals, ≥65 years, 50–64 years, and 30–49 years (from left to right)


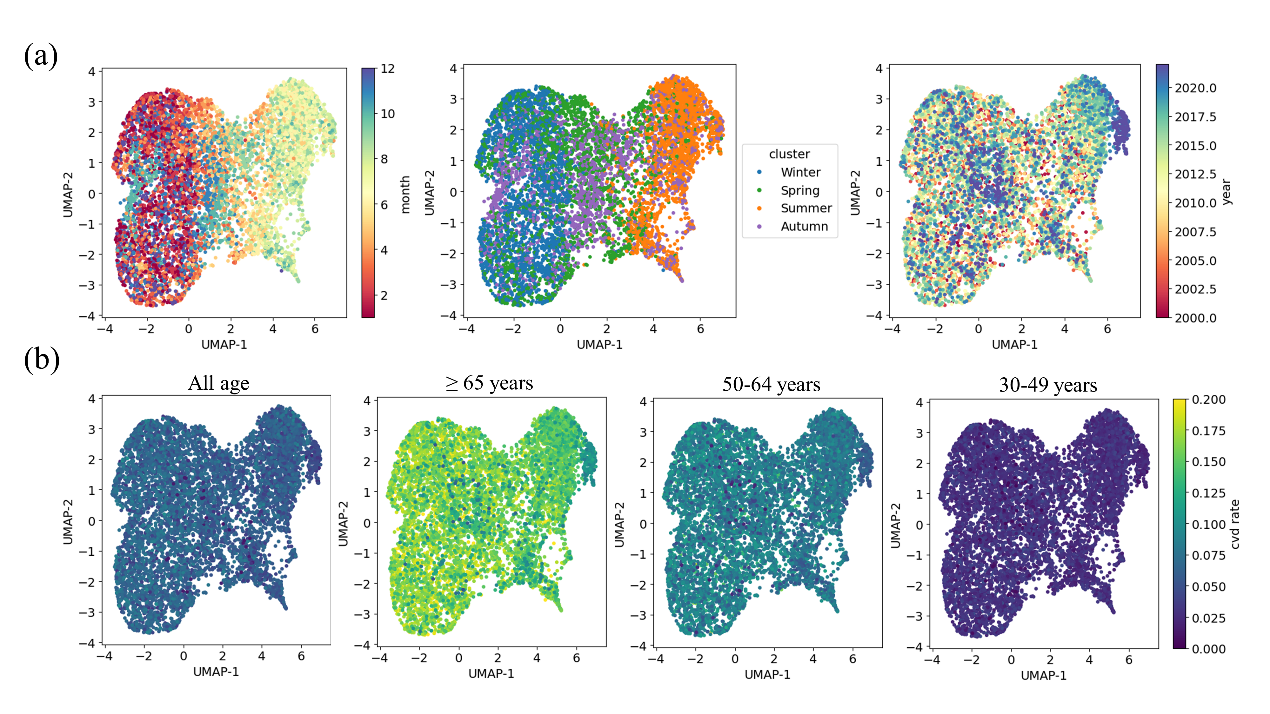


Figure S7. UMAP visualization of environmental features and CVD emergency-visit rates across Taiwan (using meteorological features). (a) UMAP projections of averaged environmental features across Taiwan, colored by month, season, and year (from left to right). (b) UMAPs colored by CVD emergency-visit rates for all individuals, ≥65 years, 50–64 years, and 30–49 years (from left to right).


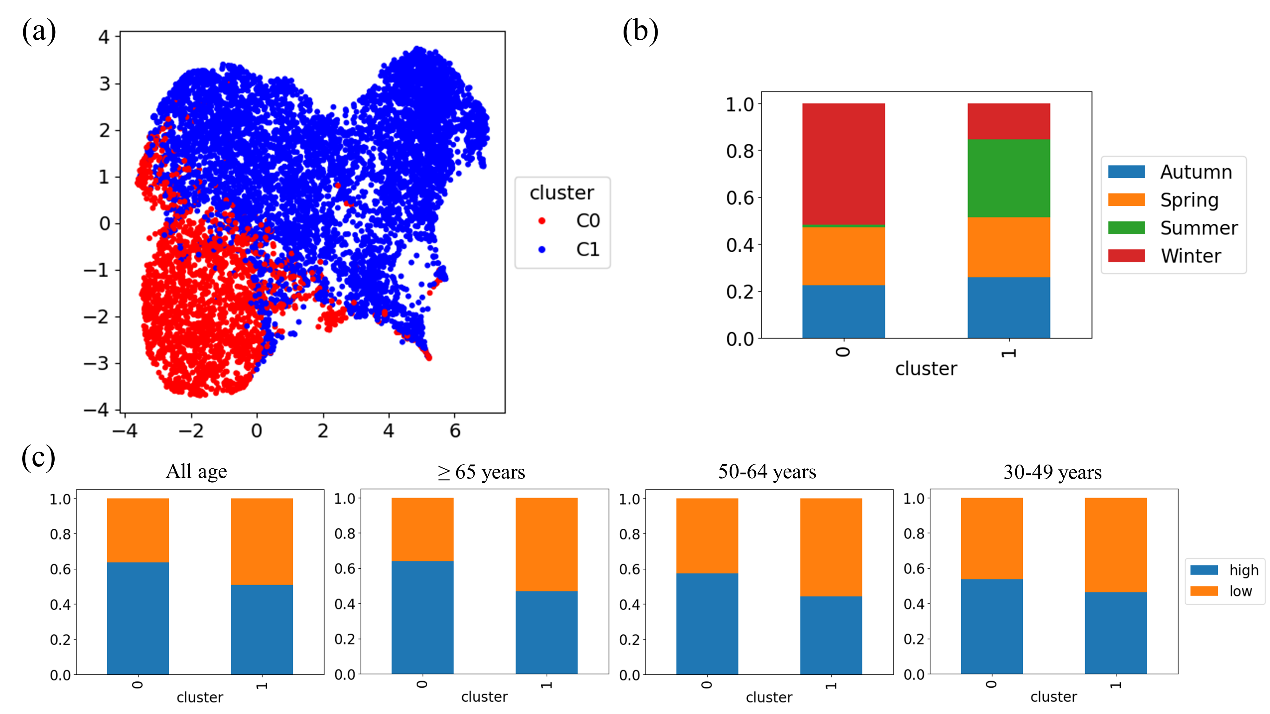


Figure S8. K-means clustering of daily samples based on environmental features across Taiwan (using meteorological features). (a) UMAP visualization showing two clusters (C0 and C1) obtained from K-means clustering (k = 2). (b) Seasonal composition of each cluster. (c) Proportions of high- and low-risk CVD days in clusters C0 and C1 for all individuals, ≥65 years, 50–64 years, and 30–49 years (from left to right)


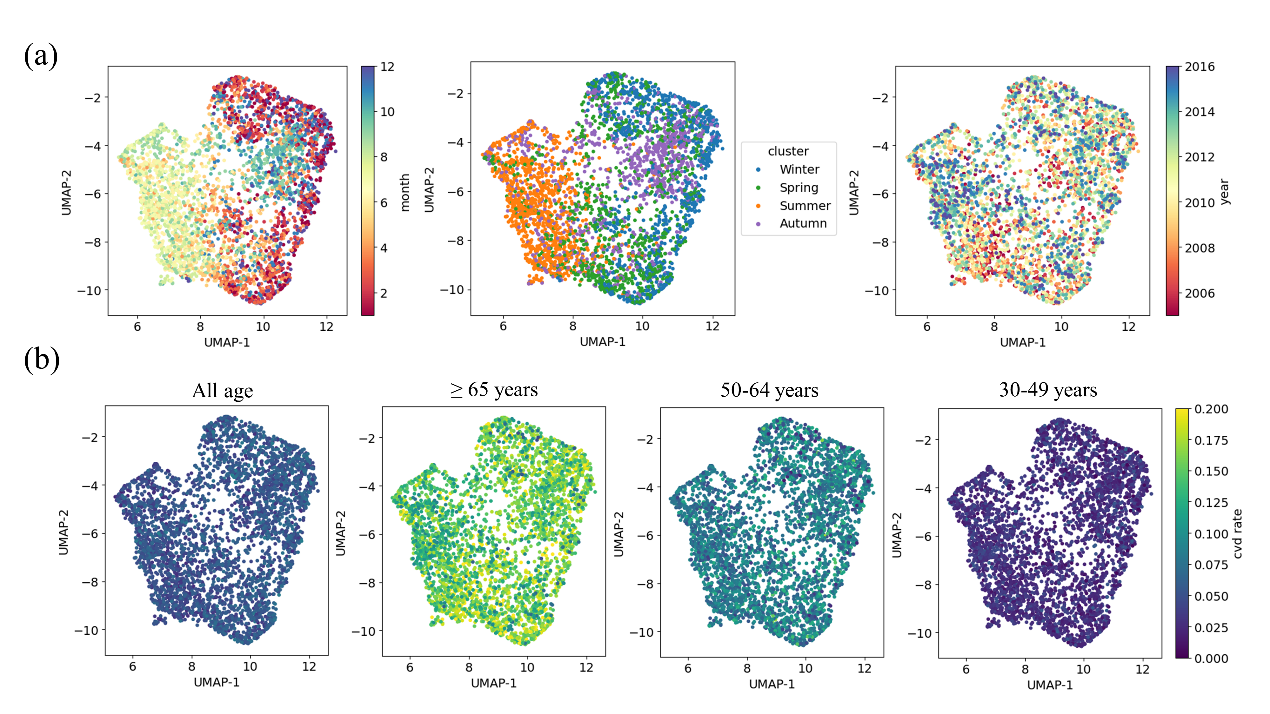


Figure S9. UMAP visualization of environmental features and CVD emergency-visit rates in the THM region. (a) UMAP projections of averaged environmental features across Taiwan, colored by month, season, and year (from left to right). (b) UMAPs colored by CVD emergency-visit rates for all individuals, ≥65 years, 50–64 years, and 30–49 years (from left to right).


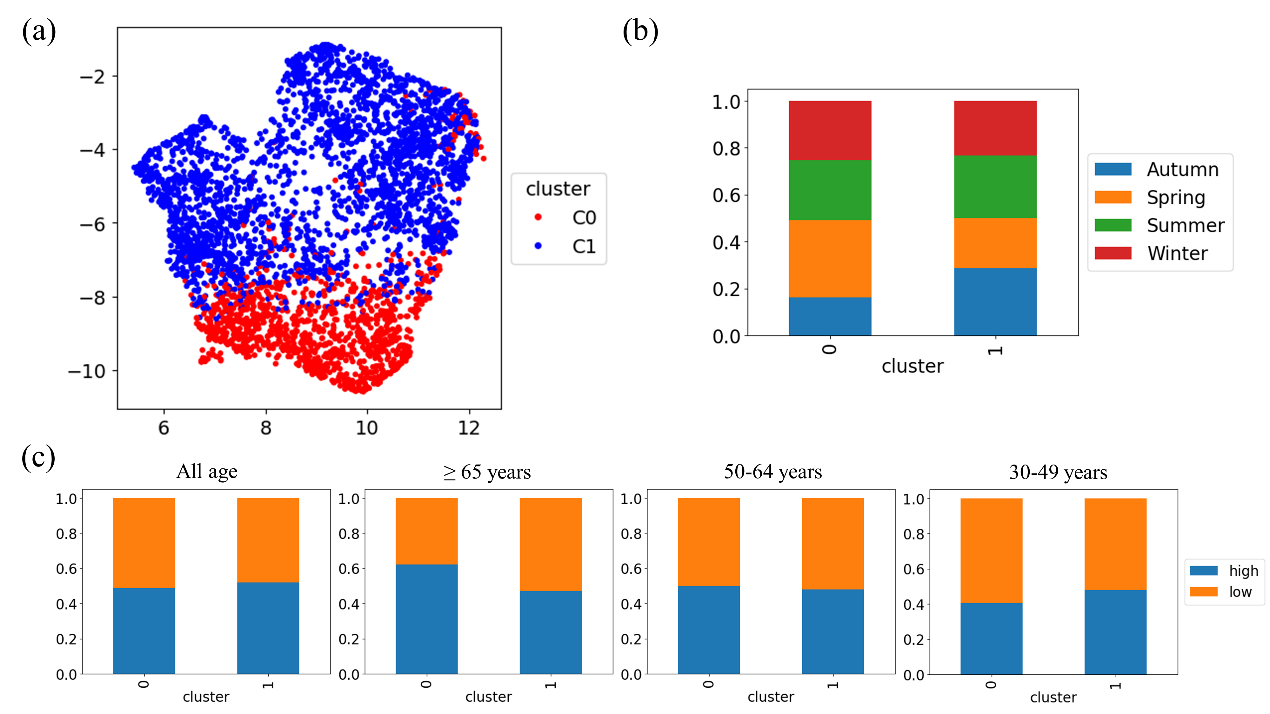


Figure S10. K-means clustering of daily samples based on environmental features in the THM region. (a) UMAP visualization showing two clusters (C0 and C1) obtained from K-means clustering (k = 2). (b) Seasonal composition of each cluster. (c) Proportions of high- and low-risk CVD days in clusters C0 and C1 for all individuals, ≥65 years, 50–64 years, and 30–49 years (from left to right)


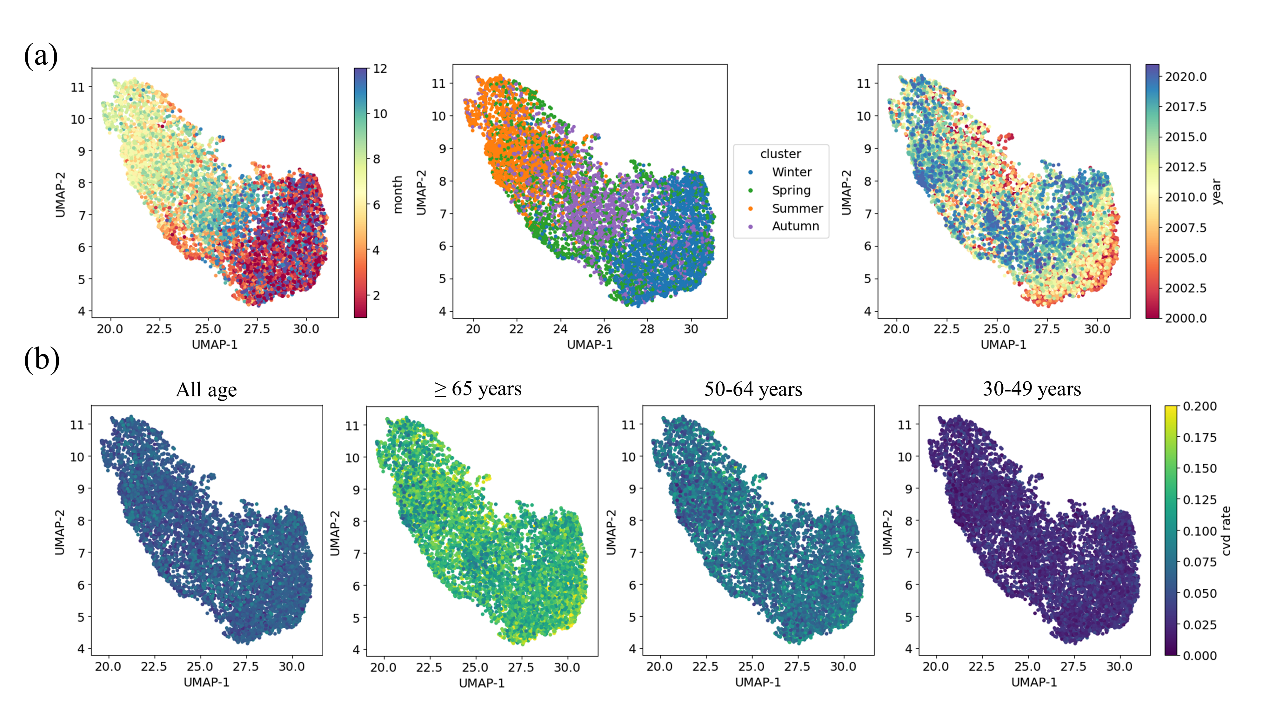


Figure S11. UMAP visualization of environmental features and CVD emergency-visit rates in the YCTKP region. (a) UMAP projections of averaged environmental features across Taiwan, colored by month, season, and year (from left to right). (b) UMAPs colored by CVD emergency-visit rates for all individuals, ≥65 years, 50–64 years, and 30–49 years (from left to right).


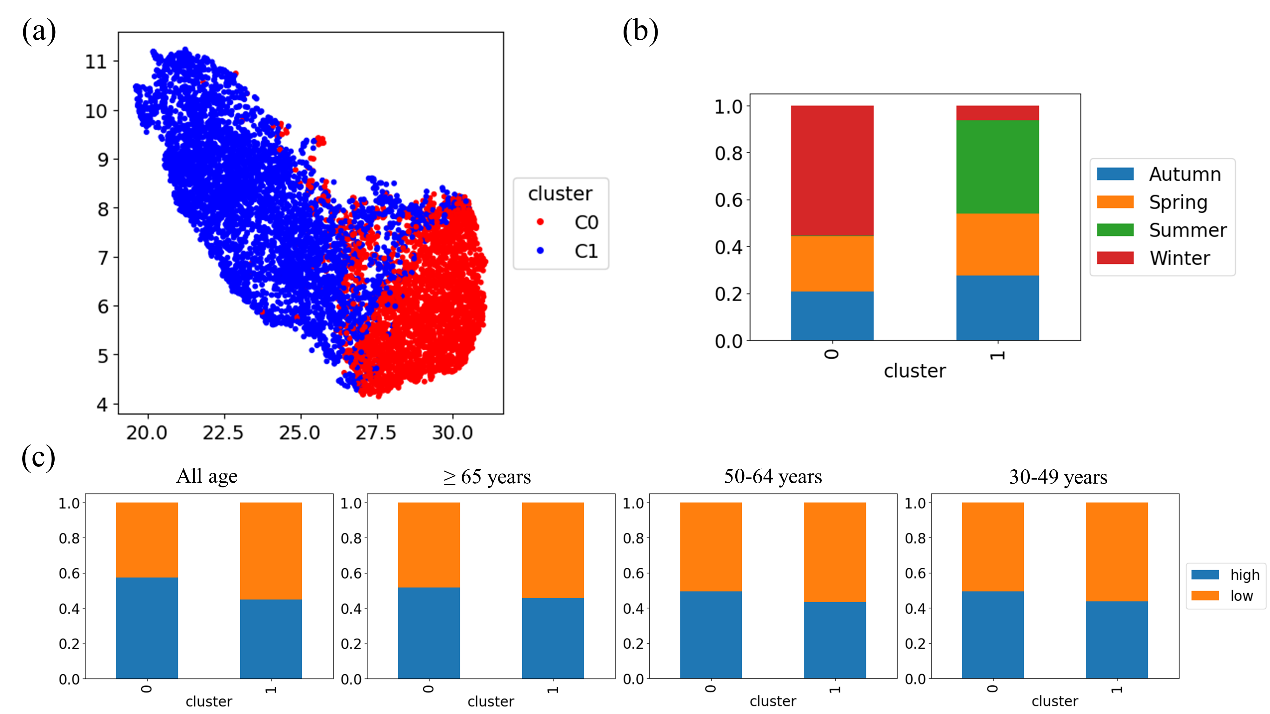


Figure S12. K-means clustering of daily samples based on environmental features in the YCTKP region. (a) UMAP visualization showing two clusters (C0 and C1) obtained from K-means clustering (k = 2). (b) Seasonal composition of each cluster. (c) Proportions of high- and low-risk CVD days in clusters C0 and C1 for all individuals, ≥65 years, 50–64 years, and 30–49 years (from left to right)


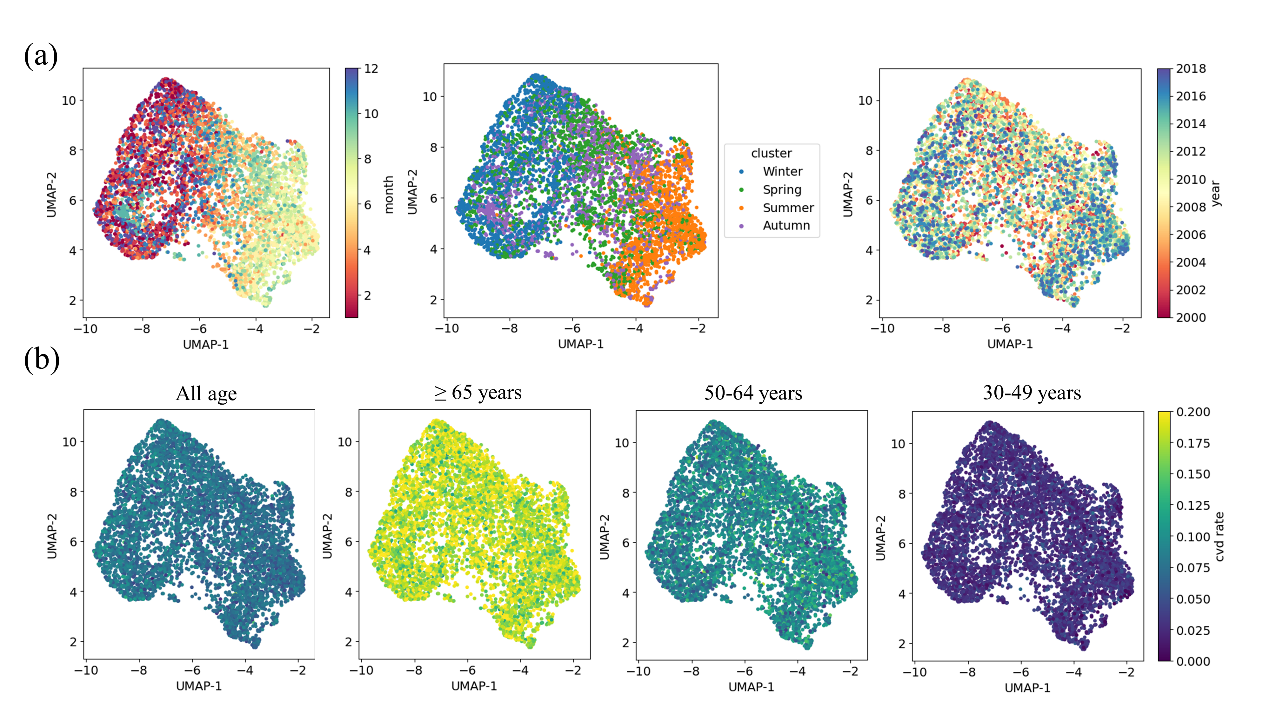


Figure S13. UMAP visualization of environmental features and CVD emergency-visit rates in the TCN region. (a) UMAP projections of averaged environmental features across Taiwan, colored by month, season, and year (from left to right). (b) UMAPs colored by CVD emergency-visit rates for all individuals, ≥65 years, 50–64 years, and 30–49 years (from left to right).


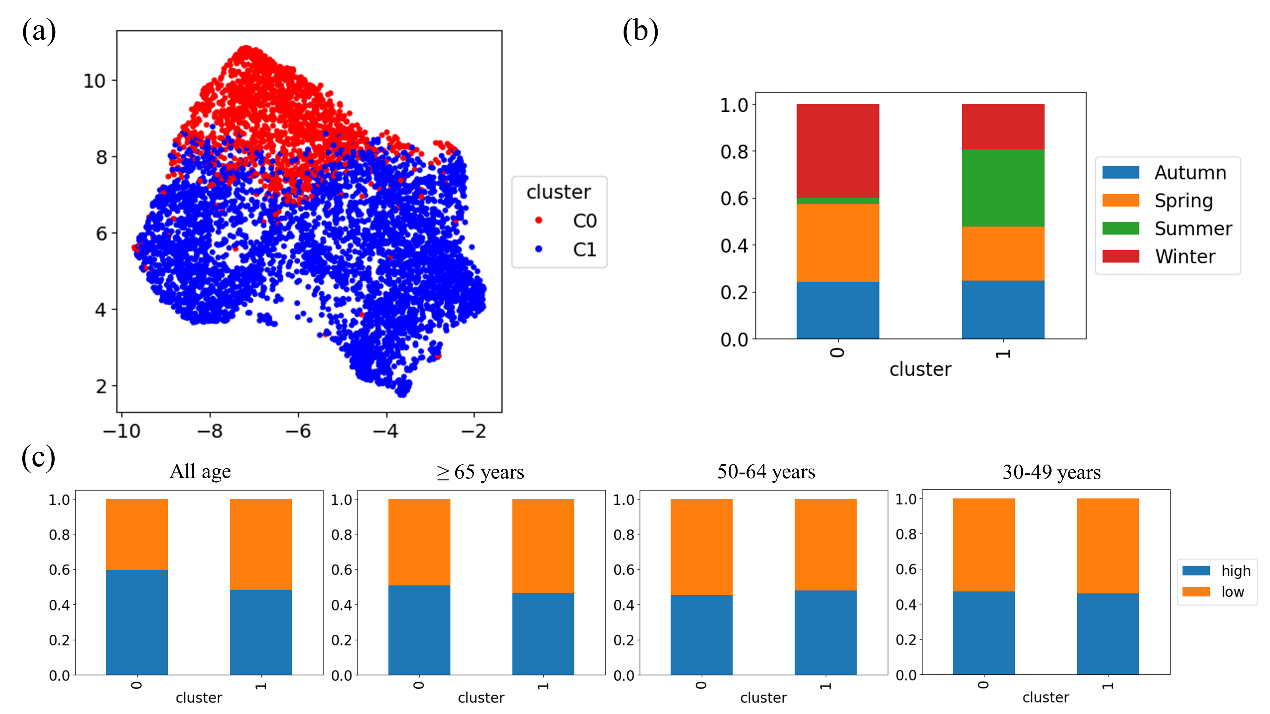


Figure S14. K-means clustering of daily samples based on environmental features in the TCN region. (a) UMAP visualization showing two clusters (C0 and C1) obtained from K-means clustering (k = 2). (b) Seasonal composition of each cluster. (c) Proportions of high- and low-risk CVD days in clusters C0 and C1 for all individuals, ≥65 years, 50–64 years, and 30–49 years (from left to right)


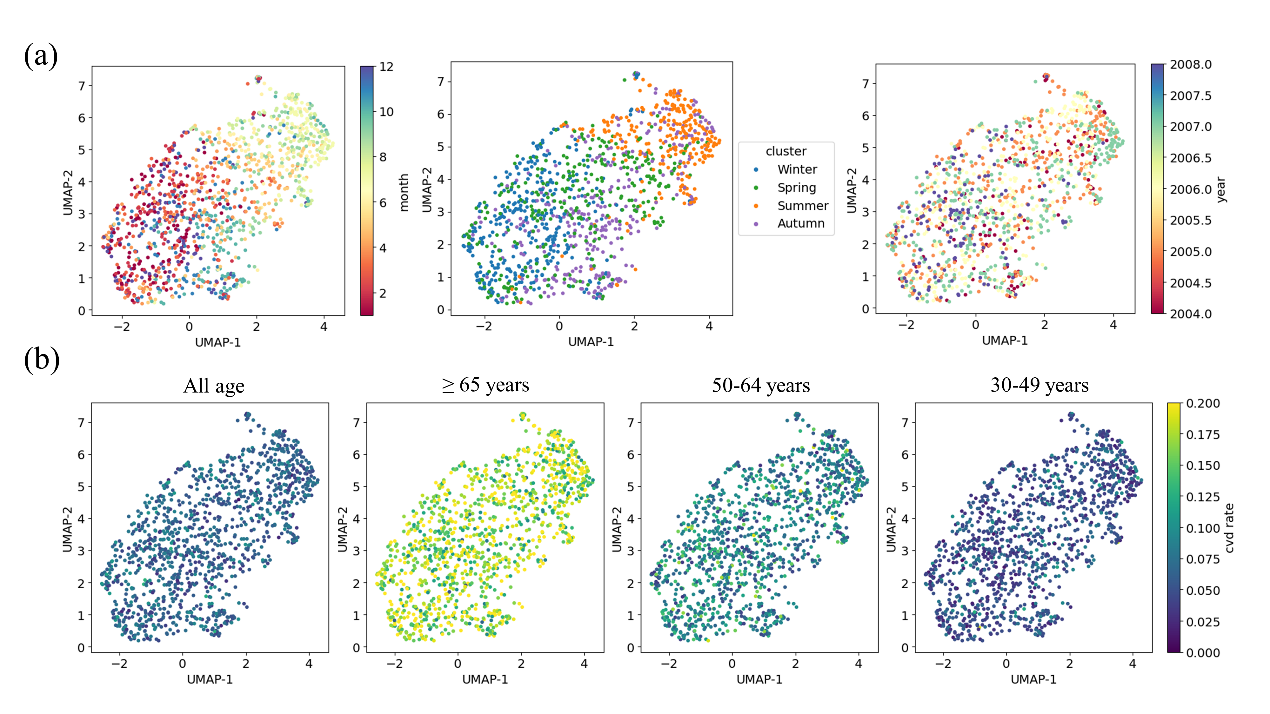


Figure S15. UMAP visualization of environmental features and CVD emergency-visit rates in the HT region. (a) UMAP projections of averaged environmental features across Taiwan, colored by month, season, and year (from left to right). (b) UMAPs colored by CVD emergency-visit rates for all individuals, ≥65 years, 50–64 years, and 30–49 years (from left to right).


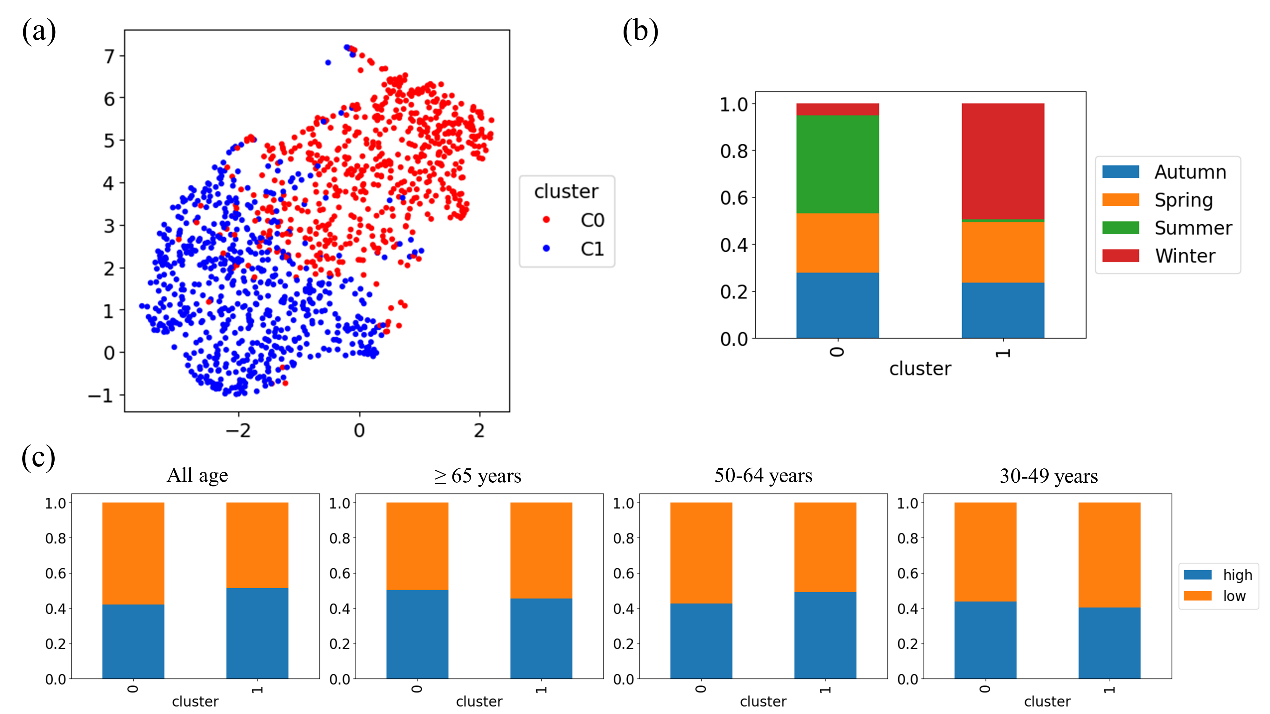


Figure S16. K-means clustering of daily samples based on environmental features in the HT region. (a) UMAP visualization showing two clusters (C0 and C1) obtained from K-means clustering (k = 2). (b) Seasonal composition of each cluster. (c) Proportions of high- and low-risk CVD days in clusters C0 and C1 for all individuals, ≥65 years, 50–64 years, and 30–49 years (from left to right)


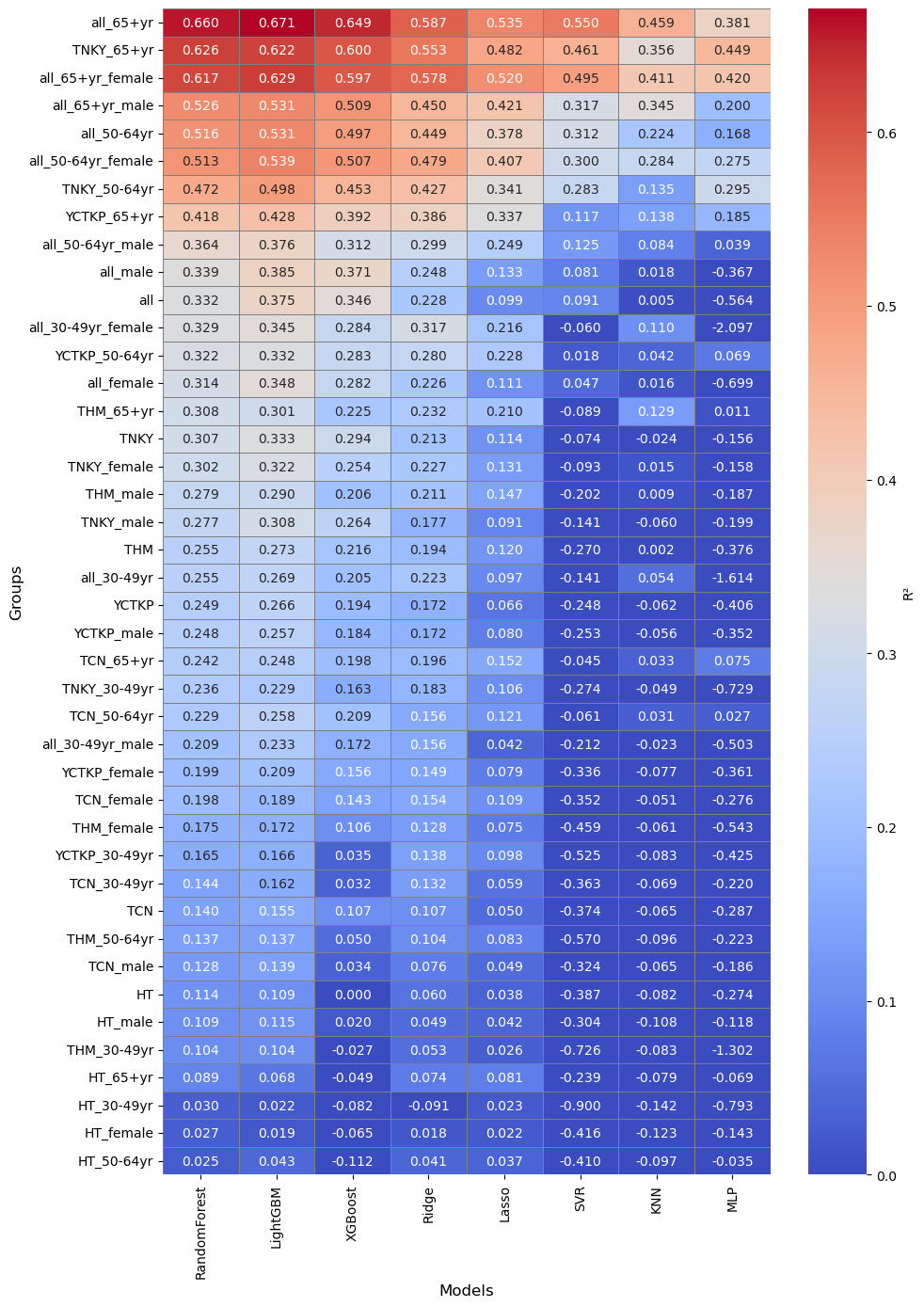


Figure S17. Heatmap of model performance (mean R²) across demographic and regional groups. Each cell represents the mean coefficient of determination (R²) between predicted and observed daily cardiovascular emergency visits (during 2000–2022) for a given model and population group. Warm colors indicate higher model predictability. Empty box represents negative R². Tree-based models (Random Forest, LightGBM, XGBoost) consistently achieved higher R² values (0.5–0.7), particularly among older adults (≥65 years) and northern Taiwan (TNKY region). Other models (Ridge, LASSO, SVR, KNN, MLP) exhibited generally lower performance, reflecting their limited ability to capture nonlinear environmental–health interactions.


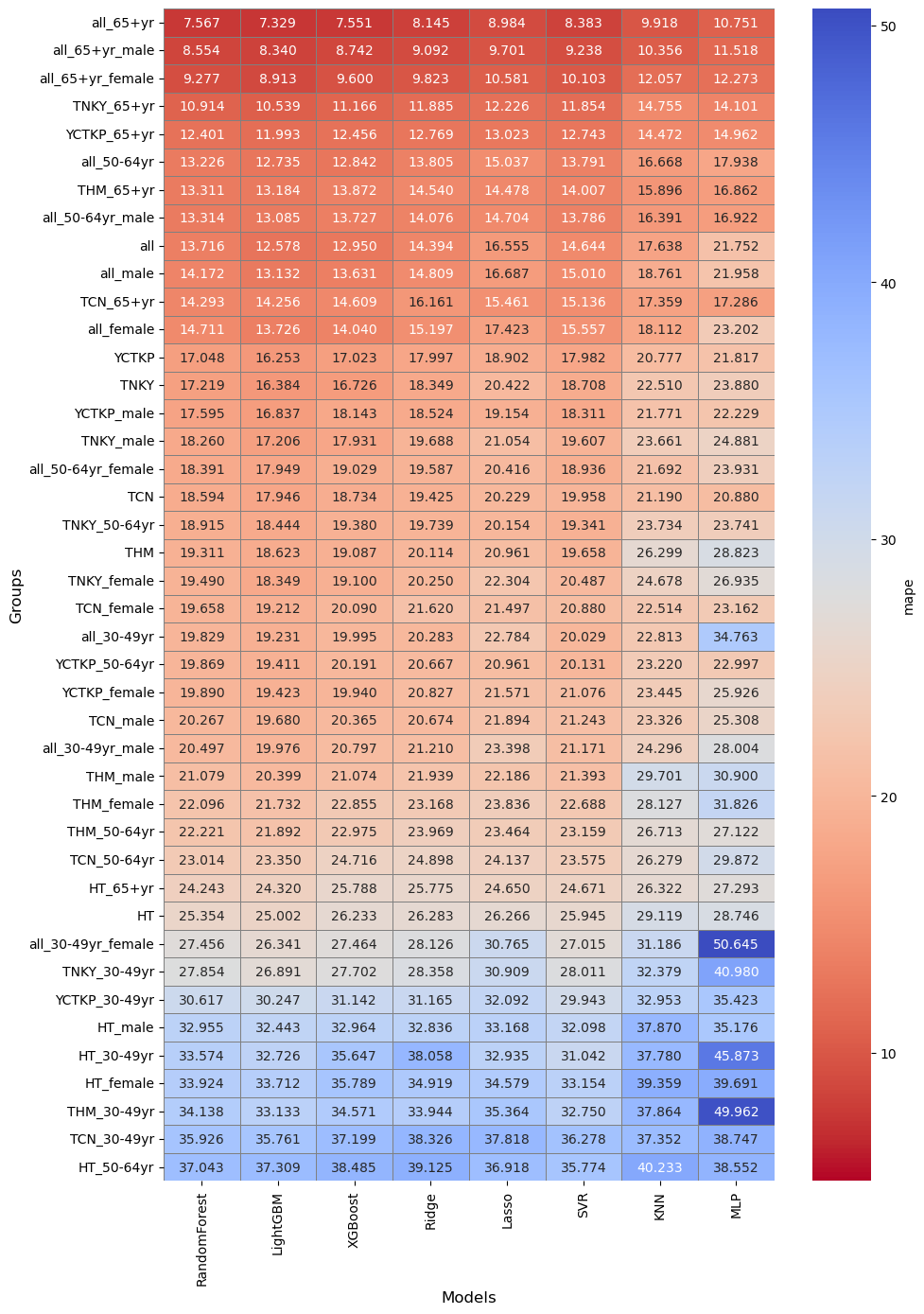


Figure S18. Heatmap of model performance (mean MAPE) across demographic and regional groups for eight machine-learning models. Each cell represents the mean daily prediction error (%) between observed and predicted cardiovascular emergency visits during 2000–2022. Cooler colors indicate smaller errors (better predictive accuracy), while warmer tones denote larger errors.
